# Doped Magnesium Ferrite Thin Films as Photoanodes for Water Oxidation

**DOI:** 10.1002/smsc.70398

**Published:** 2026-09-10

**Authors:** Tayyaba Intizar, Triet T. H. Nguyen, Kanishk Arunraj, Peter C. Sherrell, Joel van Embden, Daniel E. Gómez, Enrico Della Gaspera

**Affiliations:** ^1^ School of Science RMIT University Melbourne Victoria Australia

**Keywords:** photoelectrochemistry, photoelectrodes, solar fuels, water splitting

## Abstract

Magnesium ferrite (MgFe_2_O_4_) is a promising photoanode material for photoelectrochemical (PEC) water oxidation, combining a suitable bandgap, chemical stability, low cost, and earth abundance. However, to date, MgFe_2_O_4_ has remained largely underexplored. Here, we report the deposition of MgFe_2_O_4_ thin films via a simple aqueous route and systematically examine the effects of processing conditions and aliovalent doping on PEC performance. The fabricated films exhibit a nanocrystalline spinel structure with an optical bandgap of ~2.2 eV. We demonstrate that the PEC activity is strongly governed by annealing conditions, with high‐temperature treatment (650 °C) under nitrogen rather than air yielding optimal performance. Under these conditions, the photoanodes deliver photocurrent densities that already surpass previously reported values for MgFe_2_O_4_. Titanium doping further enhances performance, with a photocurrent of 0.14 mA/cm^2^ at 1.23 V versus reversible hydrogen electrode (RHE). Spectroscopic analyses (optical, X‐ray photoelectron spectroscopy (XPS), and impedance) link these improvements to changes in electronic structure and charge transport. Additional gains are achieved through the incorporation of a surface cocatalyst, achieving a photocurrent of 0.18 mA/cm^2^. Our results not only set the current performance record for magnesium ferrite photoanodes, establishing it as a viable PEC material, but also provide general practical guidelines for advancing ferrite‐based photoelectrodes.

## Graphical abstract

We present a simple spin‐coating method to fabricate Ti‐doped MgFe_2_O_4_ thin‐film photoanodes and demonstrate enhanced photoelectrochemical water oxidation. We reveal the role of Ti incorporation and nitrogen annealing in regulating defect states and charge transport. Ti doping narrows the bandgap and improves surface reactivity, enhancing both visible‐light absorption and photoelectrode performance.

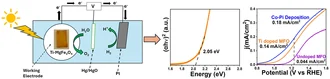

## 1 Introduction

Our current energy consumption relies heavily on fossil fuels, which are gradually depleting as the global population increases and are at risk of geopolitical supply disruption. In addition, the use of fossil fuels contributes significantly to global warming. To address growing energy needs and develop delocalized and secure energy supply, the world is transitioning toward sustainable, renewable energy sources that have limited or zero carbon dioxide emissions [1]. Solar energy stands out as the most abundant and readily available source of clean energy, making it an ideal choice for meeting future energy requirements. Photoelectrochemical (PEC) water splitting is a promising technique that uses semiconductor materials to convert solar energy into clean fuels, such as hydrogen (H_2_) and oxygen (O_2_), through photocatalysis [2]. Extensive research has focused on identifying suitable materials for PEC water splitting. The ideal materials for solar water splitting must fulfill several requirements: a bandgap between 1.6 and 2.2 eV, a conduction band edge higher than the potential for hydrogen evolution, a valence band edge lower than the redox potential for oxygen evolution, appreciable charge carrier conductivity, high stability, and excellent catalytic activity [3]. Semiconductor‐based photoanode materials, such as ZnO [4], TiO_2_ [5], WO_3_ [6], Fe_2_O_3_ [7, 8], and BiVO_4_ [9, 10], have been widely investigated for PEC water splitting. However, these materials have certain limitations, including inappropriate band positions, poor charge carrier mobility, and insufficient stability. Spinel ferrites, with the general chemical formula MFe_2_O_4_ (where M can be Zn, Cu, Mg, Ca, etc.), have attracted significant attention for PEC water splitting due to appealing properties such as low toxicity, suitable bandgap energy, chemical and thermal stability in aqueous solutions, and ease of preparation, making them ideal for producing solar fuel, such as hydrogen. Various spinel ferrites have been reported for PEC water splitting [11], such as ZnFe_2_O_4_ [12, 13, 14, 15], MnFe_2_O_4_ [16], CoFe_2_O_4_ [17], and CuFe_2_O_4_ [18]. In addition to spinel ferrites, emerging Fe‐based phases such as β‐Fe_2_O_3_ have also recently been explored for PEC water splitting due to their unique crystal and electronic structure and their potential for enhanced charge transport, offering new opportunities for the design of efficient iron‐oxide‐based photoanodes [19, 20].

Among these emerging materials, magnesium ferrite MgFe_2_O_4_ (MFO) is an n‐type semiconductor with a bandgap between 1.9 and 2.5 eV, making it a promising candidate for PEC water splitting [11]. Although MFO has been used in many applications including pseudocapacitor [21], humidity sensors [22], batteries [23], and as a magnetic material [24], magnesium ferrite remains largely unexplored for PEC application. Limited studies have shown how MFO is used within composites, rather than a standalone material, for PEC applications. For instance, Zhang et al. synthesized MgFe_2_O_4_/TiO_2_ nanocomposite via sol–gel method to enhance the efficiency of photodegradation of methylene blue [25]. Kim et al. reported a CaFe_2_O_4_/MgFe_2_O_4_ heterojunction used for isopropyl alcohol degradation and hydrogen production under visible light [26]. A 2021 study presented MFO nanostructures synthesized via hydrothermal method, achieving modest PEC water splitting (about 0.4 µA/cm^2^ at 1.23 V) [27]. This study also showed that doping MFO with zinc enhances the electronic properties of MFO by increasing electrical conductivity and reducing charge transfer resistance, thereby improving charge transport and PEC performance.

Importantly, a separate study demonstrated that incorporating titanium ions into zinc ferrite can effectively modify its structural and electronic properties, resulting in improved photocatalytic and PEC performance [28]. Titanium has been widely utilized as a dopant in various other photoanode materials, including WO_3_ [29], and Fe_2_O_3_ [30]. In ferrite systems, Ti^4+^ substitution for Fe^3+^ can modify the local electronic environment and defect chemistry to maintain charge neutrality, thereby influencing charge transport and interfacial charge‐transfer processes through enhanced charge‐transfer efficiency and possible surface‐state passivation [31]. Ti doping has also been shown to boost PEC behavior, including improved photoresponse and reduced recombination of charge carriers [28, 29, 30, 32, 33].

Despite these benefits, the effect of Ti incorporation in MFO remains unexplored. In this study, we present a simple aqueous solution spin‐coating method to deposit phase‐pure MFO films and confirm their photoactivity and then investigate for the first time titanium doping and its influence on the structure, morphology, and functional properties of MFO photoanodes.

## 2 Experimental Methods

### 2.1 Materials

Magnesium nitrate hexahydrate (Mg(NO_3_)_2_·6H_2_O, 99%), iron (III) nitrate nonahydrate (Fe(NO_3_)_3_·9H_2_O, 98%), and diammonium dihydroxybis(lactato)titanate(2‐) solution ((C_6_H_18_N_2_O_8_Ti), 50 wt.% in H_2_O, liquid) were purchased from Sigma–Aldrich. Glass slides coated with fluorine‐doped tin oxide (FTO) were purchased from NSG (TEC7, 7 Ω/sq). Milli‐Q water was used in all preparations.

### 2.2 Synthesis of MgFe_2_O_4_

MgFe_2_O_4_ thin films were deposited on an FTO glass substrate with a size of 2.5 cm x 1.5 cm by spin‐coating. First, the precursor salts were dissolved in water at an appropriate concentration (2 M for Fe(NO_3_)_3_·9H_2_O and 1 M for Mg(NO_3_)_2_·6H_2_O). The combined solution was stirred for 30 min and then sonicated for 10 min to make a homogeneous solution. Prior to deposition, the FTO glass pieces were properly cleaned with washing detergent to remove atmospheric contaminants. After that, FTO pieces were sonicated in Milli‐Q water, ethanol, acetone, and isopropanol for 10 min each to remove residual contaminants and surfactants. The MFO sol was deposited on the FTO substrate by spin coating at 3000 rpm for 36 s with acceleration set at 500 rpm/s and then stabilized on a hot plate at 500 °C for 10 min. The coated FTO pieces were then cooled down at room temperature, and the process was repeated to deposit additional layers as required. The prepared MFO thin films were subsequently annealed in air or nitrogen at different temperatures between 500 and 650 °C. Ti‐doped (MFO) were deposited using the same process, while also adding diammonium dihydroxybis(lactato)titanate(2‐) solution to the precursor solution with a nominal doping level up to 10 at.% with respect to Fe. These Ti‐doped MgFe_2_O_4_ thin films were then annealed in N_2_ at 650 °C for 2 h.

### 2.3 Characterization Techniques

The structural properties of MFO thin‐film photoanodes were examined using X‐ray diffractometry (XRD, Bruker D4 Endeavour) with Cu Kα radiation (*λ* = 1.5406 Å) operated at 40 kV and 35 mA. The resulting diffraction patterns were analyzed to identify the crystal structure and phase purity of the films. Crystallite sizes were estimated using the Debye–Scherrer equation. Surface morphology and microstructural features were characterized using scanning electron microscopy (SEM, FEI Nova 200/Verios 460L). Optical absorbance of the films was measured using a UV–vis–NIR spectrophotometer (Agilent Cary‐5000) equipped with an integrating sphere. X‐ray photoelectron spectroscopy (XPS, Thermo Scientific K‐Alpha) was employed to analyze the elemental composition, binding energies, and oxidation states of the MgFe_2_O_4_ thin films. All spectra were referenced to the C 1s peak at 284.8 eV.

The PEC performance of the MgFe_2_O_4_ thin films was evaluated using an Autolab PGSTAT204 potentiostat coupled with a solar simulator providing AM 1.5G illumination (100 mW cm^−2^). All measurements were performed in 1 M KOH (pH 13.7) as the electrolyte. Linear sweep voltammetry (LSV) was conducted to record current–potential (*J*–*V*) curves over a potential range of –0.5 to +0.8 V versus Hg/HgO at a scan rate of 10 mV s^−1^. A conventional three‐electrode configuration was employed, with the MgFe_2_O_4_ thin film as the working electrode (photoanode), a platinum rod as the counter electrode, and Hg/HgO as the reference electrode [34, 35]. All potentials were converted to the reversible hydrogen electrode (RHE) scale using the Equation (1):

(1) $$V_{\mathrm{RHE}}\;=\;V_{\mathrm{Hg}/\mathrm{HgO}}\;+\;(0.059\;\times \;\mathrm{pH})\;+\;V{{}^{\circ}}_{\mathrm{Hg}/\mathrm{HgO}\;\mathrm{vs}.\;\mathrm{SHE}}$$

where$V_{\mathrm{Hg}/\mathrm{HgO}}$is the applied bias, and $V_{\mathrm{Hg}/\mathrm{HgO}}^{{}^{\circ}}$is the standard potential of the Hg/HgO electrode versus the standard hydrogen electrode (SHE), taken as 0.12 V at 25 °C. This value was obtained by measuring the open circuit potential with a known Ag|AgCl|KCl (3 M saturated with AgCl) reference electrode, and deviates slightly from the reported value of 0.098 V [36]. Reproducibility was ensured by repeating measurements on independently prepared electrodes exhibiting the highest photocurrents.

Electrochemical impedance spectroscopy (EIS) was conducted at 1.23 V versus RHE under simulated AM 1.5G illumination to evaluate charge transfer properties. The Mott–Schottky (MS) plots were obtained by measuring the capacitance as a function of applied potential at a fixed frequency of 1 kHz in the dark, whereas the Nyquist plots were recorded by measuring the impedance over a frequency range of 10 kHz to 0.1 Hz at a fixed applied potential. The donor density (*N*_D_) and flat‐band potential (*V*_fb_) were determined by using the Equation (2) [37]:

(2) $$\frac{1}{C^{2}}=\frac{2}{\varepsilon_{0}\varepsilon_{r}A^{2}eN_{D}}\left(V-V_{\mathrm{fb}}-\frac{kT}{e}\right)$$

where C is the space‐charge capacitance, A is the illuminated area (0.45 cm^2^), $N_{D}$ is the donor density, $\varepsilon_{0}$ is the vacuum permittivity (8.854 × 10^−12^  F/m), $\varepsilon_{r}$ is the dielectric constant of MgFe_2_O_4_ (75, as reported in previous literature [38]), V is the applied potential, $V_{\mathrm{fb}}$ is the flat‐band potential, k is the Boltzmann constant (1.381 × 10^−23 ^ J/K), e is the elementary charge (1.602 × 10^−19^  C), and T is the absolute temperature.

Energy‐dispersive X‐ray spectroscopy (EDS), coupled with SEM, was employed to determine the elemental composition and confirm the presence of Mg, Fe, O, and Ti in the prepared thin films.

Cobalt phosphate (Co–Pi) cocatalyst was deposited onto the optimized photoelectrode using a photoassisted electrodeposition method adapted from previous reports [39]. A 0.1 M phosphate buffer solution (pH ≈ 7) was prepared using potassium phosphate dibasic (K_2_HPO_4_, Sigma–Aldrich, 98%) and potassium phosphate monobasic (KH_2_PO_4_, Sigma–Aldrich, 99%). Subsequently, 0.5 mM cobalt nitrate hexahydrate (Co(NO_3_)_2_·6H_2_O, Sigma–Aldrich, 98%) was added to the electrolyte and mixed thoroughly. The Co–Pi deposition was carried out by chronoamperometry at 0.1 V versus Ag/AgCl for 60 s under simulated AM 1.5G solar illumination, employing the same three‐electrode PEC configuration.

## 3 Results and Discussion

### 3.1 MgFe_2_O_4_ Films Annealed in Air

**FIGURE 1:**
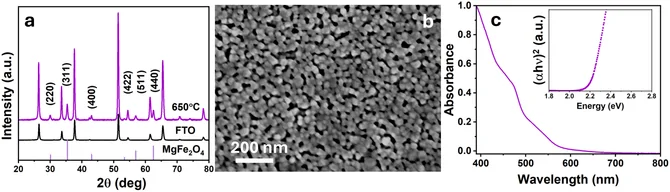
Characterization of MFO films: (a) XRD pattern. The reference peak positions according to ICDD No. 88‐1943 are shown at the bottom. (b) SEM image. (c) Absorbance spectrum (inset: Tauc plot for optical bandgap estimation).

The MFO films were prepared via spin coating from a water‐based precursor solution, followed by annealing in ambient atmosphere. The structural characteristics of the synthesized films were examined using XRD. As presented in Figure 1a, the diffraction pattern of a typical MFO thin film annealed at 650 °C for 2 h exhibits reflections that can be consistently indexed to a cubic spinel structure belonging to the Fd$\overset{¯}{3m}$ space group (ICDD No. 88‐1943). The absence of detectable secondary phases (besides the peaks of the FTO substrate) indicates that the annealing treatment is sufficient to promote complete phase formation, suggesting effective crystallization of the material without measurable impurity contributions within the instrumental detection limits. The average crystallite size, determined using Scherrer's equation based on the dominant (311) reflection at 2θ ~35.4°, was estimated to be approximately 20 nm. The nanoscale crystallite dimensions suggest a high density of grain boundaries, which may influence charge separation and interfacial transport dynamics. In thin‐film photoelectrodes, reduced crystallite size can be beneficial by providing increased reaction sites and by shortening carrier diffusion lengths. However, the increased density of grain boundaries may also introduce recombination centers, highlighting a trade‐off between bulk and interfacial recombination processes [40, 41, 42]. For completeness, the temperature‐dependent evolution of the diffraction patterns is provided in the Supporting Information (Figure S1), showing phase‐pure crystalline films at all investigated temperatures.

Figure 1b illustrates the surface morphology of a representative MFO thin film. The SEM micrograph reveals a porous, polycrystalline architecture with homogeneous surface coverage. Porous films are particularly relevant for PEC applications because of the increased amount of accessible reaction sites compared to dense, compact coatings. The average particle size estimated from SEM analysis (23.5 ± 5.0 nm) closely matches the crystallite size derived from XRD measurements. This agreement implies that the grains are predominantly monocrystalline, suggesting minimal internal grain subdivision. SEM images of films annealed at different temperatures (Figure S2) reveal a progressive grain growth with increasing annealing temperature. This trend is expected and reflects thermally driven atomic diffusion and grain boundary migration. The evolution of grain size with temperature is expected to influence both electrical conductivity and charge transport behavior, thereby impacting the overall PEC response. The optical absorption characteristics of the MFO thin films are shown in Figure 1c. A well‐defined absorption edge is observed near 550 nm, indicating light absorption within the visible spectral region. The optical bandgap, estimated via Tauc analysis, was determined to be approximately 2.21 eV, in close agreement with previously reported values [26, 43, 44]. The consistency of the bandgap across the investigated annealing temperatures (Figure S3 and Table S1) suggests that thermal treatment does not introduce substantial alterations to the intrinsic electronic structure. Instead, annealing primarily governs structural evolution rather than band structure modification.

**FIGURE 2:**
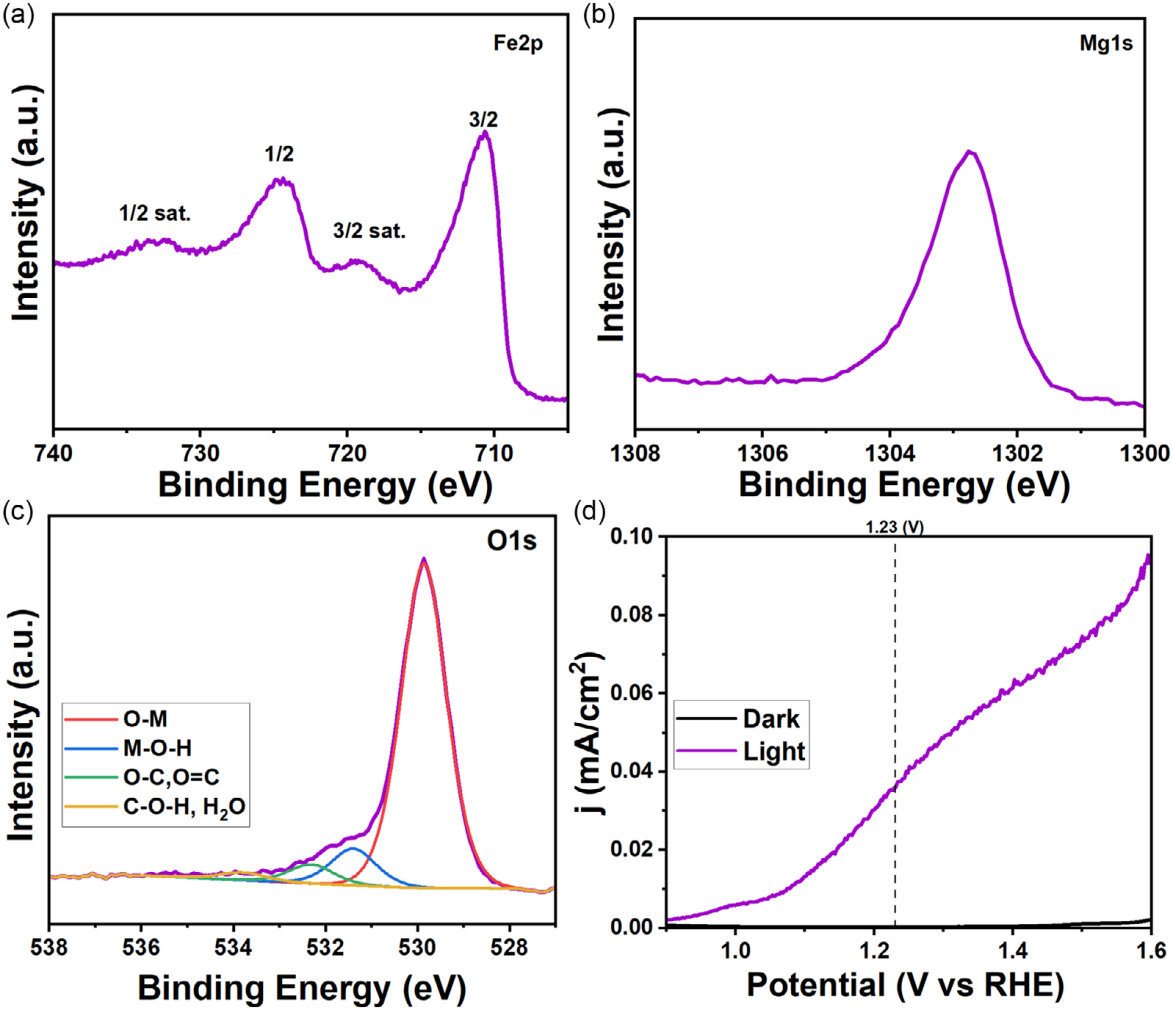
(a–c) XPS analysis in the (a) Fe 2p, (b) Mg 1s, and (c) O 1s regions for MFO films annealed at 650 °C. (d) LSV curves for MFO annealed at 650 °C in the dark (black line) and under simulated sunlight (purple line) in 1 M KOH.

The chemical states of the constituent elements were investigated using XPS, as presented in Figure 2a–c. The Fe 2p core‐level spectrum (Figure 2a) displays two prominent peaks at 710.63 and 724.58 eV, corresponding to the Fe 2p_3/2_ and Fe 2p_1/2_ transitions, respectively, accompanied by weak satellite features. The observed spectral characteristics are consistent with Fe^3+^ species typically reported for spinel ferrites, indicating that iron predominantly exists in the trivalent oxidation state. The stabilization of Fe^3+^ is essential for maintaining charge neutrality within the MgFe_2_O_4_ lattice and reflects the expected cationic configuration of the spinel structure. The Mg 1s spectrum (Figure 2b) exhibits a single, well‐defined peak centered at 1303.87 eV, consistent with Mg^2+^ species incorporated within the oxide framework. The absence of additional components or peak asymmetry suggests a uniform chemical environment for magnesium, supporting its successful integration into the host lattice. Such chemical stability implies minimal surface segregation or secondary phase formation, which is consistent with the phase purity inferred from XRD analysis. The O 1s spectrum (Figure 2c) can be fitted with two contributions located at approximately 529.8 and 532.0 eV. The lower binding energy component is assigned to lattice oxygen associated with metal–oxygen bonding in the spinel structure, while the higher binding energy feature is attributed to a series of surface‐related species, including hydroxyl groups and adsorbed oxygen‐containing contaminants. The relative presence of these components reflects the typical surface chemistry of metal oxide thin films exposed to ambient conditions. Importantly, the dominance of the lattice oxygen contribution indicates preservation of the oxide framework following annealing. Overall, the XPS results confirm the expected oxidation states for iron and magnesium, supporting the formation of MFO. These observations are also consistent with previous reports on spinel ferrites [11].

Following confirmation of phase‐pure MFO formation, the PEC performance of the air‐annealed films was evaluated to assess their photoactivity. Figure 2d presents the LSV measured in 1 M KOH electrolyte. Among the samples investigated, the films annealed at 650 °C exhibit the highest photocurrent density of approximately 0.036 mA cm^−2^ at 1.23 V versus RHE. While the absolute photocurrent remains relatively modest, the observed value exceeds previously reported results for undoped MFO thin films [1, 27], indicating that the adopted synthesis and annealing strategy contributes to measurable performance enhancement. The improved photoresponse at 650 °C can be rationalized by considering the structural and morphological evolution induced by thermal treatment. XRD analysis indicates enhanced crystallinity at elevated annealing temperatures, which is expected to improve charge carrier transport by reducing structural disorder and minimizing defect‐induced scattering. Concurrently, SEM observations reveal a porous microstructure that favors interfacial contact between the electrode and the electrolyte. This combination of improved lattice ordering and accessible surface morphology is likely to facilitate more efficient photogenerated charge separation and interfacial charge‐transfer processes. The superior performance of the 650 °C film suggests the presence of an optimal balance between crystallization and microstructural preservation. The dependence of the PEC behavior on the annealing temperature and on the thickness of the MFO coatings is provided in the Supporting Information (Figure S4). Notably, the three‐layer film annealed at 650 °C exhibits the highest photocurrent density, suggesting that film thickness also plays a critical role in governing light absorption and charge transport dynamics.

### 3.2 Effect of Annealing in Inert Atmosphere

**FIGURE 3:**
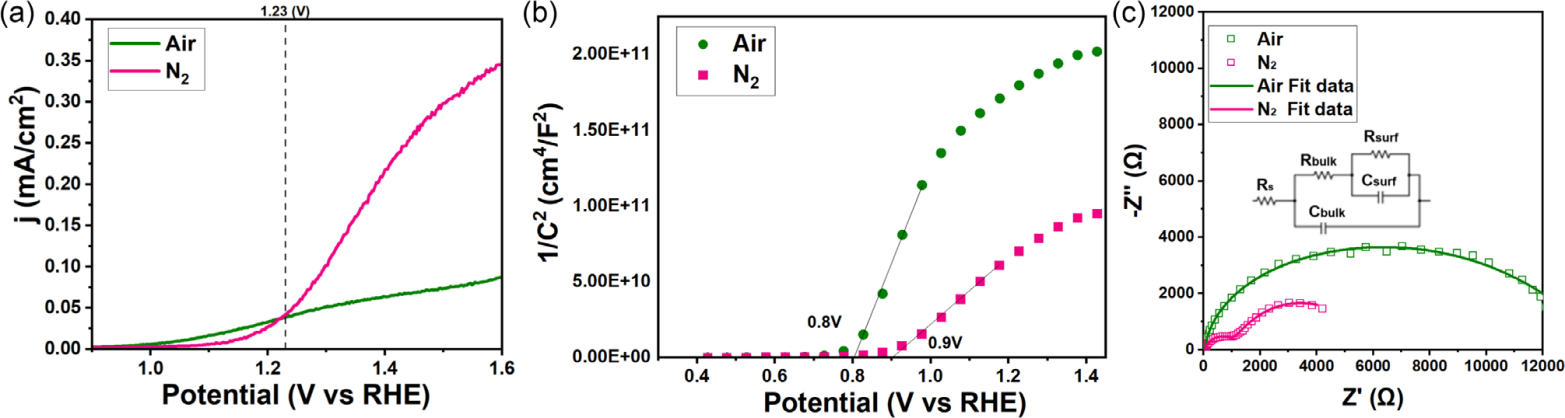
(a) LSV under simulated sunlight, (b) Mott–Schottky plot, and (c) Nyquist plot for air and nitrogen N_2_ annealed MFO at 650 °C.

An effective strategy to enhance the PEC performance of some photoanode materials involves modification of the annealing atmosphere, which can strongly influence defect chemistry, electronic transport, and interfacial processes [15, 45]. Figure 3a presents the PEC response of MFO thin films annealed under a nitrogen atmosphere at 650 °C. The nitrogen‐annealed films exhibit clear photoactivity, with the three‐layer sample delivering a photocurrent density of approximately 0.044 mA cm^−2^ at 1.23 V versus RHE under AM 1.5 illumination in 1 M KOH electrolyte. Although the value recorded at this specific potential represents only a moderate increase relative to the corresponding air‐annealed films treated at the same temperature, the photocurrent–potential behavior undergoes a notable transformation. Specifically, the nitrogen‐annealed films display a steeper rise in photocurrent and significantly enhanced response at more positive applied potentials. This behavior suggests that nitrogen annealing modifies the charge transport characteristics of the films, promoting more efficient extraction of photogenerated charges. Importantly, the altered slope of the photocurrent curves indicates a reduction in transport‐related losses, while the pronounced enhancement at higher potentials suggests mitigation of recombination pathways associated with poor carrier mobility [46]. These results demonstrate that nitrogen annealing primarily influences electronic transport dynamics, yielding a more substantial performance enhancement under higher operating voltages.

To further elucidate the influence of nitrogen annealing, the structural, optical, and surface characteristics of MFO thin films treated under nitrogen at different temperatures were examined, with the corresponding results provided in the Supporting Information (Figures S5, S6, S7, and Table S2). These analyses reveal behavior comparable to that observed for air‐annealed films, indicating that the basic properties remain largely unaffected by the annealing atmosphere, with the exception of a slight reduction in crystallite/particle size and related increase in grain density. Instead, the enhanced PEC response of nitrogen‐annealed films is more plausibly associated with subtle alterations in defect chemistry or surface‐related electronic properties [45, 47, 48]. For example, annealing under an oxygen‐deficient environment is known to promote the formation of oxygen‐related defects, particularly oxygen vacancies, which can introduce donor‐like states and enhance electronic conductivity in metal oxide systems [42, 46]. As such, EIS was performed to examine the charge transport and interfacial properties of air‐ and nitrogen‐annealed MFO thin films treated at 650 °C (Figure 3b,c).

The MottS plots (Figure 3b) exhibit a positive slope, confirming the n‐type semiconducting character of magnesium ferrite. The flat‐band potential (*V*_fb_) shifts slightly from ~0.8 V for the air‐annealed film to ~0.9 V following nitrogen annealing. Although oxygen‐deficient annealing is often associated with increased donor density through the formation of oxygen vacancies, the resulting flat‐band potential does not necessarily shift cathodically as it is also influenced by surface states, defect distribution, and Fermi‐level pinning at the semiconductor/electrolyte interface [49]. Therefore, the slight anodic shift observed here likely reflects changes in the interfacial electronic structure rather than solely the increase in donor concentration. Nevertheless, nitrogen annealing results in a substantial increase in donor density (*N*_D_), rising from 3.61 × 10^18^ cm^−3^ (air) to 9.53 × 10^18^ cm^−3^ (nitrogen).

The elevated donor density implies enhanced electronic conductivity, which supports improved bulk charge transport and explains the steeper photocurrent rise and higher photocurrent densities at elevated applied potentials. The Nyquist plots (Figure 3c) further corroborate this behavior. The nitrogen‐annealed film exhibits a reduced semicircle diameter, indicating lower charge‐transfer resistance (*R*_ct_). By applying a fitting procedure with a suitable equivalent circuit (inset of Figure 3c), the electrical resistances associated with different electron‐transfer processes during the PEC reactions were estimated [45]. Specifically, for air‐annealed MFO, the electron‐transfer resistances for the bulk (*R*_bulk_) and the surface (*R*_surf_) were determined to be 1600 and 4800 Ω cm^2^, respectively. For nitrogen‐annealed MFO, on the other hand, these values decreased significantly to 490 Ω cm^2^ (*R*_bulk_) and 2100 Ω cm^2^ (*R*_surf_). The pronounced reduction in bulk resistance indicates that the inert‐gas thermal treatment primarily enhances the bulk conductivity of MFO, likely by reducing structural defects and improving charge transport pathways. This improvement in electrical properties is expected to directly contribute to enhanced PEC performance. Therefore, the increased donor density and reduced resistance indicate that nitrogen annealing predominantly improves the electronic transport properties of MFO, which accounts for the enhanced PEC performance, especially at higher applied bias. A comparative evaluation of the structural, optical, and chemical characteristics of MFO thin films annealed in air and nitrogen at the same temperature is provided in the Supporting Information (Figure S8). The results reveal no significant differences in crystal structure, optical properties, or chemical environment (oxidation state) of the elements, suggesting that these parameters are not responsible for the enhanced PEC performance of nitrogen‐annealed MFO. To further clarify the origin of this enhancement, the electrochemically active surface area (EASA) of air‐ and nitrogen‐annealed films was evaluated (Figure S9). The comparable EASA values (double layer capacitance is 29.6 µF/cm^2^ for air annealing and 40.2 µF/cm^2^ for nitrogen annealing) indicate that the observed photocurrent enhancement is not likely to be governed by surface area effects. The charge separation efficiency in the bulk ($\eta_{\mathrm{bulk}}$) and charge transfer efficiency on the surface ($\eta_{\mathrm{surf}}$), calculated from the photocurrent measured in the presence and in the absence of a hole scavenger, and from the optical absorption spectra [15], show that nitrogen annealing slightly improves the bulk of the material with marginal effects on the surface (Table S3). The reproducibility of our deposition method was further assessed for the optimized photoanodes (three‐layer films annealed at 650 °C). The photocurrent density measured at 1.23 V versus RHE exhibited high consistency, yielding an average value of 0.043 ± 0.001 mA cm^−2^, with a maximum value of 0.044 mA cm^−2^ (Figure S10). Importantly, high reproducibility is also observed at high potentials, with average photocurrents of 0.29 ± 0.01 mA cm^−2^ measured at 1.5 V versus RHE. These results confirm the reliability of the fabrication process and the stability of the PEC response.

### 3.3 Titanium Doping

**FIGURE 4:**
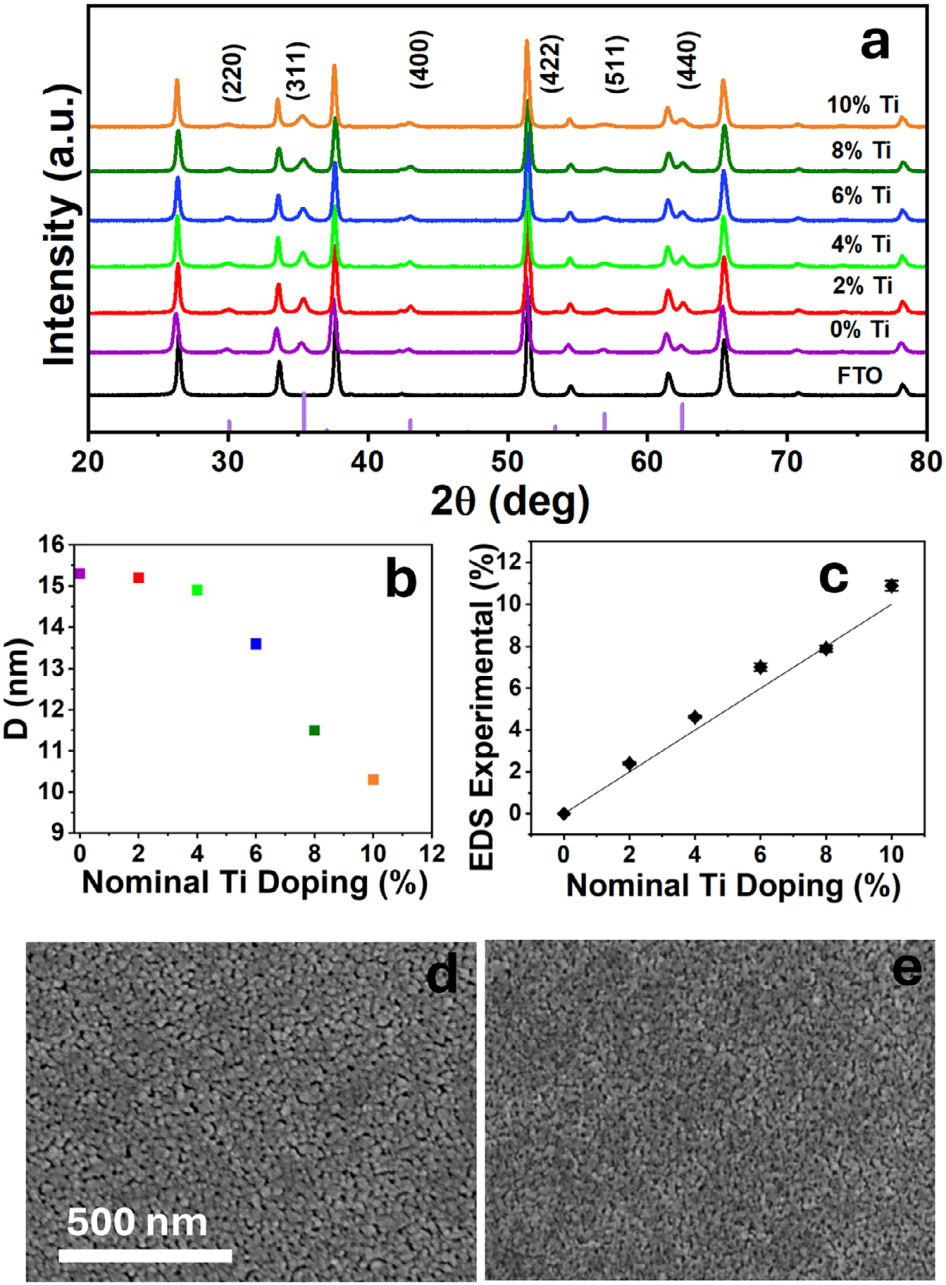
(a) XRD patterns of Ti‐doped MFO thin films with varying Ti concentration. (b) Variation of crystallite size as a function of Ti doping. (c) Correlation between nominal Ti doping concentration and measured Ti content from EDS analysis. (d) SEM image of undoped MFO. (e) SEM image of 10% Ti‐doped MFO.

Despite the measurable photoactivity, the relatively modest photocurrent motivated the exploration of additional strategies for enhancing PEC performance of MFO films. In this context, titanium was selected as a dopant to tailor the electronic and transport properties of MFO. Figure 4a presents the XRD patterns of Ti‐doped MFO thin films annealed at 650 °C. All samples exhibit diffraction peaks characteristic of the cubic spinel structure, consistent with the reference pattern. The absence of secondary phases across the investigated Ti concentrations indicates that Ti incorporation does not disrupt the host lattice, confirming successful dopant accommodation while preserving phase purity.

The crystallite sizes, estimated from the (311) reflection using Scherrer's equation, are summarized in Figure 4b. A systematic decrease in crystallite size with increasing Ti concentration is observed. This behavior is attributed to dopant‐induced lattice distortions arising from Ti^4+^ substitution for Fe^3+^, which can modify crystallization kinetics and suppress grain growth. The presence of Ti may also hinder atomic diffusion, limiting crystallite coalescence. Similar effects have been reported in other Ti‐doped ferrite systems [50, 51]. EDS analysis was performed to verify the elemental composition and Ti incorporation in the doped MFO thin films. The analysis confirms the presence of Mg, Fe, O, and Ti across all samples in the expected amount based on stoichiometry. Importantly, the measured Ti concentrations closely match the nominal doping levels (Figure 4c), indicating reliable dopant control during synthesis. No detectable impurity elements were observed within the instrumental resolution, supporting the chemical purity of the films. The surface morphology of undoped and Ti‐doped MFO thin films is presented in Figure 4d,e, with additional images provided in the Supporting Information (Figure S11). All films exhibit uniform polycrystalline microstructures. Increasing Ti concentration leads to a noticeable reduction in particle size and a progressively more compact morphology, particularly at higher doping levels. This trend is consistent with the crystallite size reduction observed in XRD analysis, indicating that Ti incorporation influences grain growth behavior.

**FIGURE 5:**
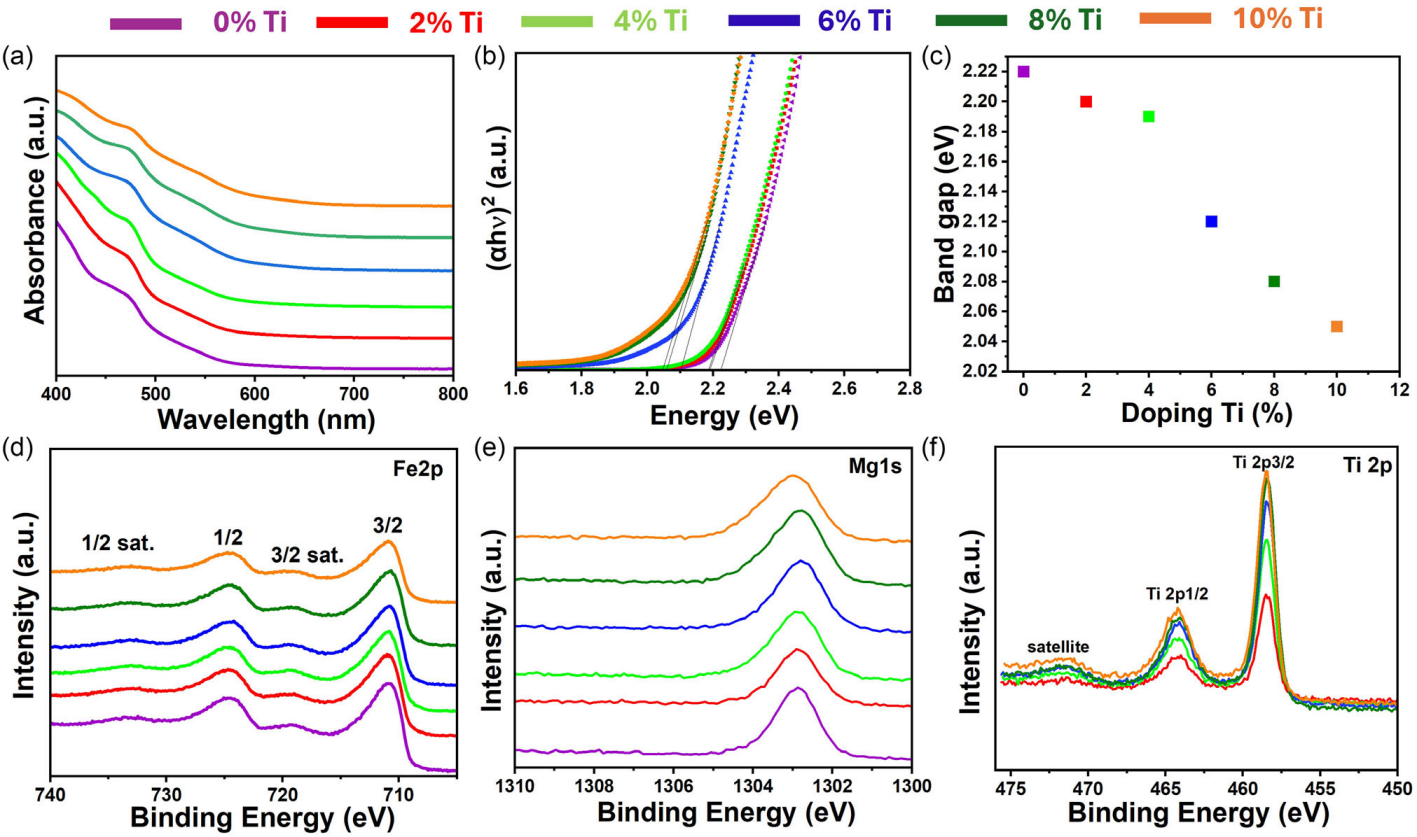
Optical and XPS analysis of Ti‐doped MFO: (a) UV–vis absorbance spectra, (b) Tauc plots, (c) variation of direct bandgap with Ti content, and (d–f) XPS spectra of Fe 2p (d), Mg 1s (e), and Ti 2p (f).

The optical properties of undoped and Ti‐doped MFO thin films were examined using UV–vis spectroscopy, as shown in Figure 5a. All samples exhibit an absorption onset in the 580–600 nm range, which is consistent with the characteristic optical response of spinel ferrites. As shown in Figure 5b,c, the optical bandgap decreases progressively with increasing Ti concentration. This bandgap narrowing is attributed to dopant‐induced modification of the electronic structure associated with Ti substitution at Fe sites, consistent with previous reports on Ti‐doped ferrite systems.

Bandgap values estimated from Tauc analysis are summarized in Figure 5c. The observed trend aligns with previous reports on Ti‐doped ferrite systems, where dopant incorporation modifies the electronic states and influences optical transitions, potentially impacting PEC behavior. A summary of the crystallite size, optical bandgap, and particle size of Ti‐doped MFO thin films at different Ti concentrations is provided in Table S4.

The incorporation of Ti into the MFO lattice was further examined using XPS (Figure 5d–f). The Fe 2p spectrum exhibits characteristic Fe^3+^ features, with peaks at 710.63 and 724.58 eV. The Mg 1s peak appears at 1303.87 eV, consistent with Mg^2+^ species. Both of these are in line with the results presented earlier for undoped films.

In the doped samples, the Ti 2p spectrum displays peaks at 458.30 eV (2p_3/2_) and 464.04 eV (2p_1/2_), confirming that titanium is predominantly present as Ti^4+^, consistent with previous reports [28]. The absence of detectable secondary crystalline phases is supported by the XRD results (Figure 4a), which show only the characteristic reflections of spinel MgFe_2_O_4_. The intensity of the Ti peaks is also seen to progressively increase with the doping % as confirmation of the higher amount of Ti detected in these samples. The survey, O 1s, and valence band spectra are provided in the Supporting Information (Figure S12). Interestingly, although only minimal variations in the valence band edge are detected, considering also the decrease in bandgap energy with Ti doping, the Fermi energy is seen to progressively shift upward toward the CB in Ti‐doped samples, suggesting a more pronounced n‐type character (Table S4).

**FIGURE 6:**
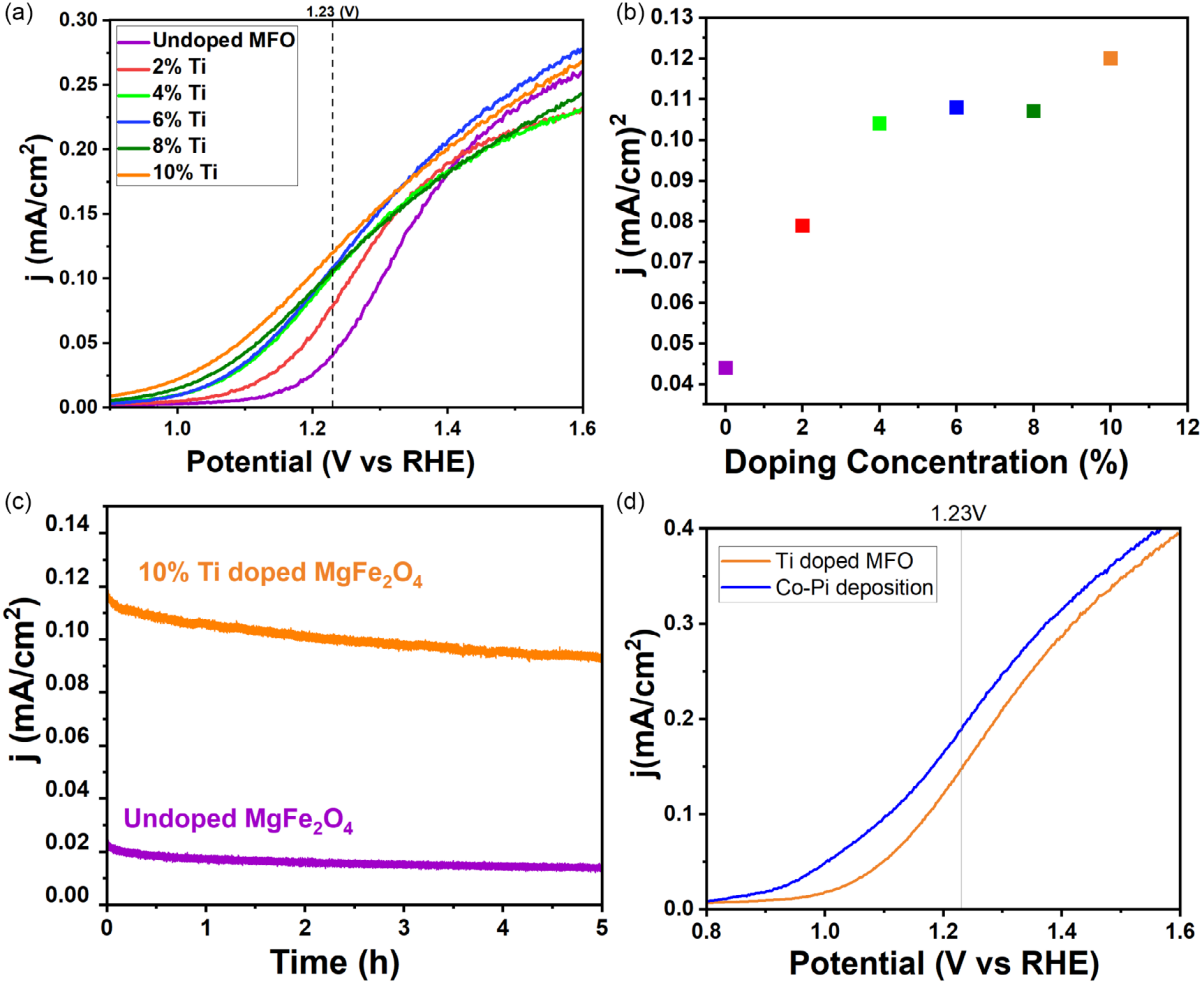
Photoelectrochemical performance of Ti‐doped MFO: (a) LSV plots under illumination, (b) photocurrent density at 1.23 V versus RHE as a function of Ti doping concentration obtained from the representative LSV measurements shown in (a), (c) chronoamperometric stability measurements at 1.23 V versus RHE for undoped and 10% Ti‐doped MFO, and (d) LSV curves of 10% Ti‐doped MFO before and after Co–Pi electrodeposition, showing an increase in LSV photocurrent density from ~0.14 to ~0.18 mA cm^−2^.

The PEC performance of undoped and Ti‐doped MFO thin films was evaluated in 1 M KOH at 1.23 V versus RHE under simulated solar illumination (Figure 6a). A systematic increase in photocurrent density is observed with increasing Ti concentration. Undoped MFO films exhibit a photocurrent density of approximately 0.044 mA cm^−2^, whereas Ti doping at 10 at.% increases the photocurrent density to ~0.14 mA cm^−2^, corresponding to a ~threefold enhancement (Figure 6b). The enhanced photoresponse is consistent with the structural and optical modifications induced by Ti incorporation, as discussed earlier. Calculations of $\eta_{\mathrm{bulk}}$ and $\eta_{\mathrm{surf}}$ highlight that Ti doping is mainly effective in improving the surface charge transfer efficiency rather than affecting the bulk properties of MFO (Table S3). Collectively, the results indicate that Ti doping effectively enhances the PEC behavior of MFO by modifying its electronic and transport‐related properties. Reproducibility was evaluated for the optimized 10 at.% Ti‐doped MFO films (three‐layer configuration and 650 °C annealing). The measurements yielded consistent results, with an average photocurrent density of 0.135 ± 0.013 mA cm^−2^ and a maximum value of ~0.14 mA cm^−2^ (Figure S13), confirming the reliability of the fabrication process. The operational stability of undoped and 10 at.% Ti‐doped MFO films was also evaluated using chronoamperometry at 1.23 V versus RHE over a 5 h period (Figure 6c). Both samples exhibit reasonably stable photocurrent responses under continuous illumination. The Ti‐doped film consistently maintains higher photocurrent densities than the undoped sample throughout the measurement. The Ti‐doped MFO also shows better stability than undoped MFO, with a smaller photocurrent decay over 5 h (~24% drop compared to over 40% reduction for the undoped sample), confirming improved operational durability. The gradual decrease in photocurrent observed for both samples during long‐term operation can be attributed to surface‐related deactivation processes commonly observed in metal oxide photoanodes under alkaline conditions [52]. These include partial surface reconstruction, accumulation of reaction intermediates, and gradual blocking of active sites by adsorbed oxygenated species during the oxygen evolution reaction [53]. Such processes can reduce the efficiency of hole transfer at the semiconductor–electrolyte interface over extended operation. These results demonstrate that Ti incorporation enhances PEC performance without compromising operational stability, highlighting its effectiveness for improving ferrite‐based photoanodes.

EASA analysis of 10 at.% Ti‐doped MFO thin films (Figure S14) reveals that Ti incorporation does not significantly alter the active surface area compared to undoped MFO. The capacitive current profiles as a function of scan rate indicate nearly identical electrochemically accessible areas (the double layer capacitance is 40.2 µF/cm^2^ for undoped and 27.7 µF/cm^2^ for 10% Ti doped, so the active area could be slightly lower in Ti‐doped samples), suggesting that the improvements in PEC performance observed with Ti doping are not due to enhanced surface area. EIS measurements for undoped and 10 at.% Ti‐doped MFO thin films (Figure S15) provide further insight into electronic modifications induced by Ti doping. MottS analysis shows that the undoped MFO electrode exhibits a donor density of 9.53 × 10^18^ cm^−3^ with a flat band potential of approximately 0.90 V, whereas the 10% Ti‐doped MFO sample shows a slightly reduced donor density of 7.23 × 10^18^ cm^−3^, accompanied by a cathodic shift of the flat band potential to 0.64 V. The decrease in donor density suggests that Ti doping does not increase the free carrier concentration but instead modifies the defect chemistry and electronic structure of the material. This behavior can be rationalized by considering charge‐compensating defect chemistry in the spinel lattice. Although Ti^4+^ substitution at Fe^3+^ sites is generally expected to act as donor doping, the incorporation of higher‐valence Ti can also alter the native defect equilibrium, particularly by reducing the concentration of oxygen vacancies that otherwise contribute to n‐type conductivity. In this case, charge compensation may occur through the annihilation or suppression of oxygen vacancy defects rather than by increasing free electron concentration. As a result, the net donor density can decrease despite Ti incorporation, accompanied by an increase in bulk resistance. Therefore, the observed electrical response suggests that Ti doping primarily modifies the defect landscape rather than acting as a conventional shallow donor [54, 55, 56]. It should be noted that the MottS model assumes that the space charge capacitance is negligible compared to the Helmholtz capacitance, which may not be strictly valid for these nanostructured electrodes. Therefore, the extracted flat‐band potentials should be regarded as qualitative indicators rather than absolute values [7]. Nevertheless, the significant cathodic shift provides qualitative evidence of a favorable change in the band energetics at the semiconductor/electrolyte interface, consistent with enhanced band bending and improved charge separation. This behavior is consistent with the PEC measurements, where the Ti‐doped electrode displays an earlier onset potential and higher photocurrent density. Collectively, these results imply that the improved PEC performance primarily originates from favorable band energetics and more efficient interfacial charge transfer rather than an increase in donor density. Nyquist analysis reveals a significant redistribution of the resistive components upon Ti doping (Figure S15). The undoped MFO electrode exhibits a bulk resistance of ~490 Ω cm^2^ and a substantially higher surface (charge–transfer) resistance of ~2100 Ω cm^2^. In contrast, the 10 at.% Ti‐doped film shows a bulk resistance of ~750 Ω cm^2^, accompanied by a pronounced reduction in surface resistance to ~300 Ω cm^2^. Although a moderate increase in bulk resistance is observed, the dramatic decrease in surface resistance dominates the overall impedance response, resulting in a lower effective interfacial resistance. The substantial reduction in charge‐transfer resistance indicates markedly improved interfacial charge‐transfer kinetics, in agreement with the measurements conducted in the presence of hole scavenger. These findings suggest that the enhanced PEC performance of Ti‐doped MgFe_2_O_4_ primarily originates from improved interfacial processes rather than bulk transport modifications alone. To further enhance the surface reaction kinetics, a Co–Pi cocatalyst was deposited on the best‐performing Ti‐doped MFO via electrodeposition [39]. A cathodic shift is observed after cocatalyst deposition, with a photocurrent density increased to ~0.18 mA cm^−2^ at 1.23 V (and 0.37 mA cm^−2^ at 1.5 V), as shown in Figure 6d, demonstrating that surface cocatalyst modification accelerates water oxidation by facilitating hole transfer at the semiconductor–electrolyte interface. The stability of the Co–Pi‐modified photoelectrode was also assessed, showing a slight decrease in performance over time, similarly to the doped MFO; however, the higher performance compared to the unmodified photoelectrode is still maintained, indicating that Co–Pi enhances the photocurrent while also providing a sustained photocurrent response under chronoamperometric conditions (Figure S16). It is important to note that the performance during chronoamperometric measurement at 1.23 V is lower than the best value obtained from the LSV measurement because LSV captures the photocurrent during a single potential scan, while chronoamperometry reflects the sustained photocurrent under continuous illumination at a fixed potential. Importantly, characterization post extended testing using XRD, SEM, and XPS (Figure S17) shows no modification of the material, suggesting reasonable operational stability. In detail, the XPS spectra retained the characteristic Fe, Mg, Ti, O, Co, and P signals, confirming the presence of the Co–Pi catalyst layer after the stability test and the absence of chemical modifications of the MFO layer. SEM and XRD also confirm that both the morphology and structure of the photoanode remained largely preserved during PEC testing.

Our results highlight that a combination of nitrogen annealing, Ti doping, and Co–Pi surface modification in magnesium ferrites synergistically improves both bulk and surface charge transport, enhances charge separation, and accelerates surface reactions, resulting in significantly superior PEC performance. This detailed understanding of bulk and surface electronic properties provides valuable guidance for rational design of high‐performance MFO‐based photoanodes.

## 4 Conclusion

We have presented a synthesis method to deposit high‐quality magnesium ferrite thin films and demonstrated their record PEC performance for water oxidation. We conducted a systematic investigation to evaluate the influence of annealing atmosphere and doping on the structural, optical, and PEC properties of magnesium ferrite photoanodes. The results demonstrate that nitrogen annealing preserves the crystal structure, bandgap, and oxidation states of the elements within the material while significantly modifying its electronic transport characteristics. Nitrogen‐annealed MFO films exhibit enhanced PEC performance, achieving a photocurrent density of ~0.044 mA cm^−2^ at 1.23 V versus RHE in 1 M KOH, superior to that of air‐annealed samples. EIS reveals that this improvement primarily originates from defect‐mediated conductivity changes and reduced interfacial resistance rather than bulk structural modifications. Further improvement was achieved through Ti doping. Ti incorporation maintains phase purity while inducing bandgap narrowing. The optimized 10 at.% Ti‐doped MFO films deliver a photocurrent density of 0.14 mA cm^−2^, far superior to that of undoped MFO. Electrochemical analysis confirms that the performance improvement is dominated by a substantial reduction in charge‐transfer resistance, highlighting the critical role of interfacial charge transport. Additional performance gains were obtained through the deposition of a cobalt phosphate OER surface cocatalyst, yielding a record photocurrent density of 0.18 mA cm^−2^ at 1.23 V versus RHE. Collectively, these findings establish magnesium ferrite as an emerging photoanode material for water oxidation and demonstrate that defect engineering via annealing atmosphere and aliovalent doping is an effective strategy for tailoring its PEC response.

## Funding

This work was supported by the Australian Research Council (DP220100020 and DP230101764).

## Conflicts of Interest

The authors declare no conflicts of interest.

## Supporting information

Supplementary Material

## Data Availability

The data that support the findings of this study are available from the corresponding author upon reasonable request.
