# Supplementary material for: Doped Magnesium Ferrite Thin Films as Photoanodes for Water Oxidation
Source: Small Sci. 2026 Sep 10;6(9):e70398. doi: 10.1002/smsc.70398 (PMC13574032; doi:10.1002/smsc.70398)
Supplement: Supplementary file 1 [file SMSC-6-e70398-s001.pdf]

Supporting Information

Doped Magnesium Ferrite Thin Films as Photoanodes for Water Oxidation

Tayyaba Intizar[1], Triet T. H. Nguyen[1], Kanishk Arunraj[1], Peter Sherrell[1], Joel van Embden[1], Daniel E. Gómez[1], Enrico Della Gaspera[1,]*

[1] School of Science, RMIT University, Melbourne VIC 3000, Australia.

Corresponding author: *enrico.dellagaspera@rmit.edu.au

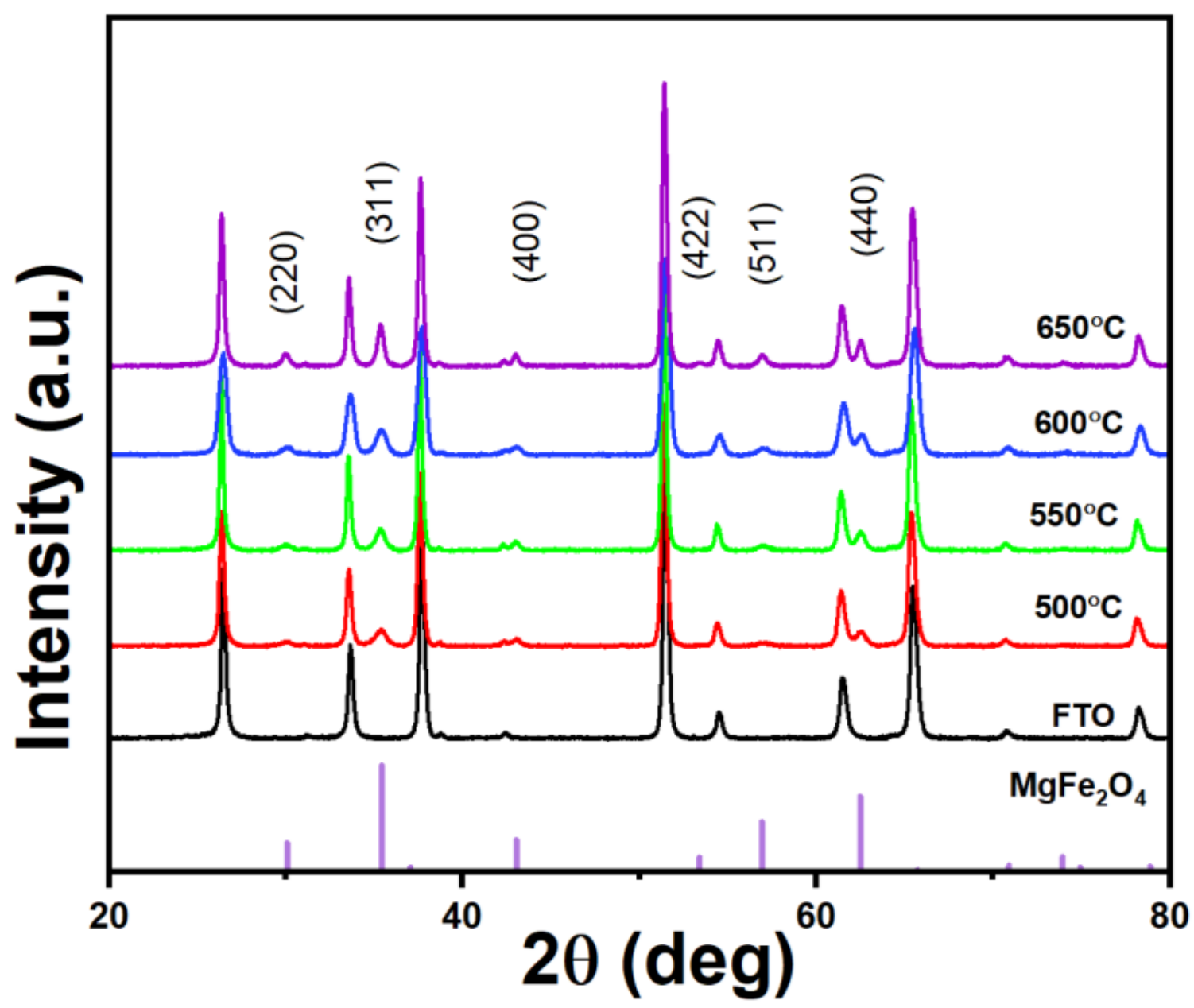

Figure S1. X-ray diffraction (XRD) patterns of $MgFe_2O_4$ thin films annealed in air at 500, 550, 600, and 650 °C for 2 h. The reference peak positions for spinel magnesium ferrite according to ICDD No. 88-1943 are shown at the bottom.

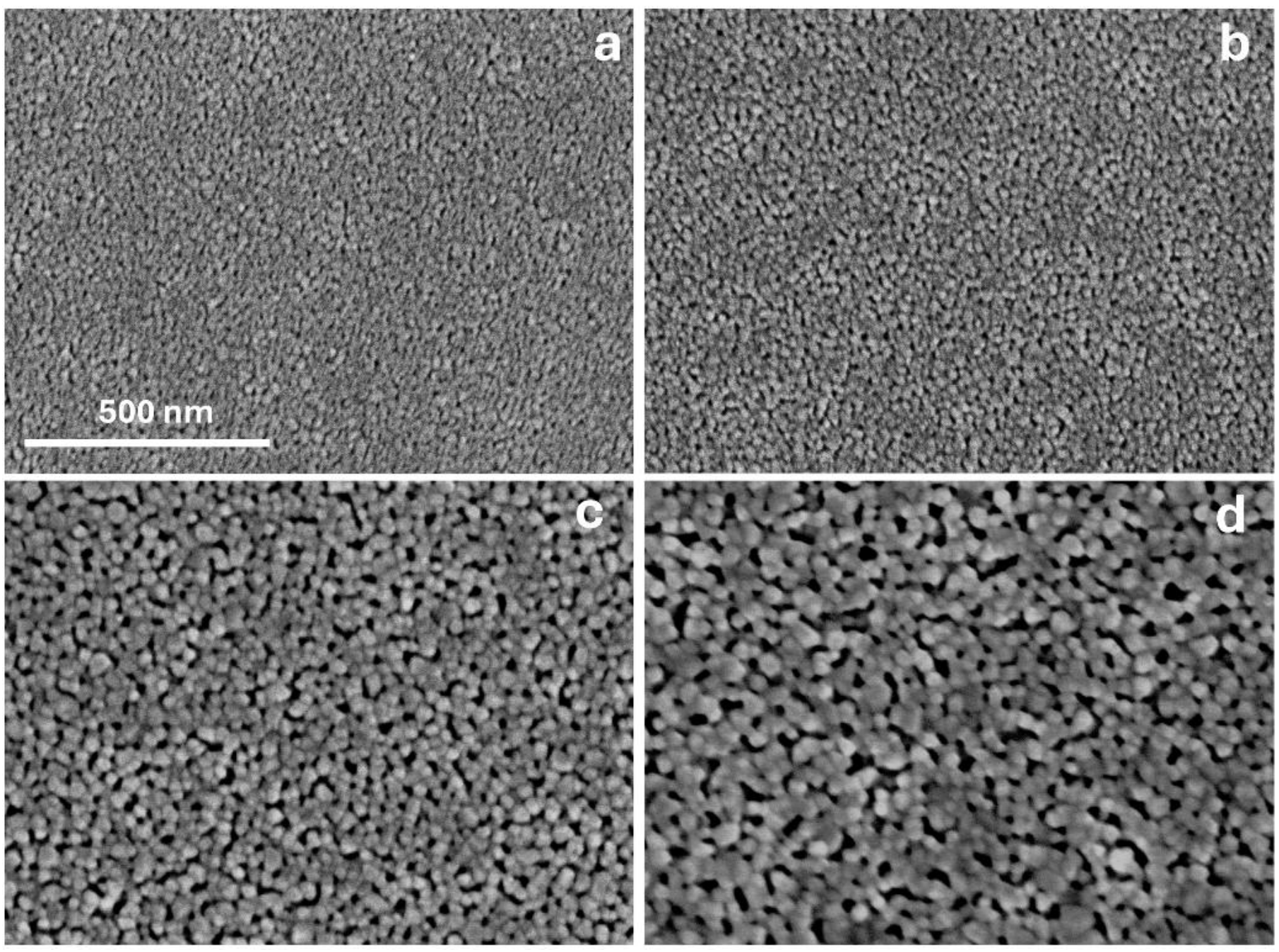

Figure S2. SEM images of air-annealed $MgFe_2O_4$ thin films treated at (a) 500, (b) 550, (c) 600, and (d) 650 °C.

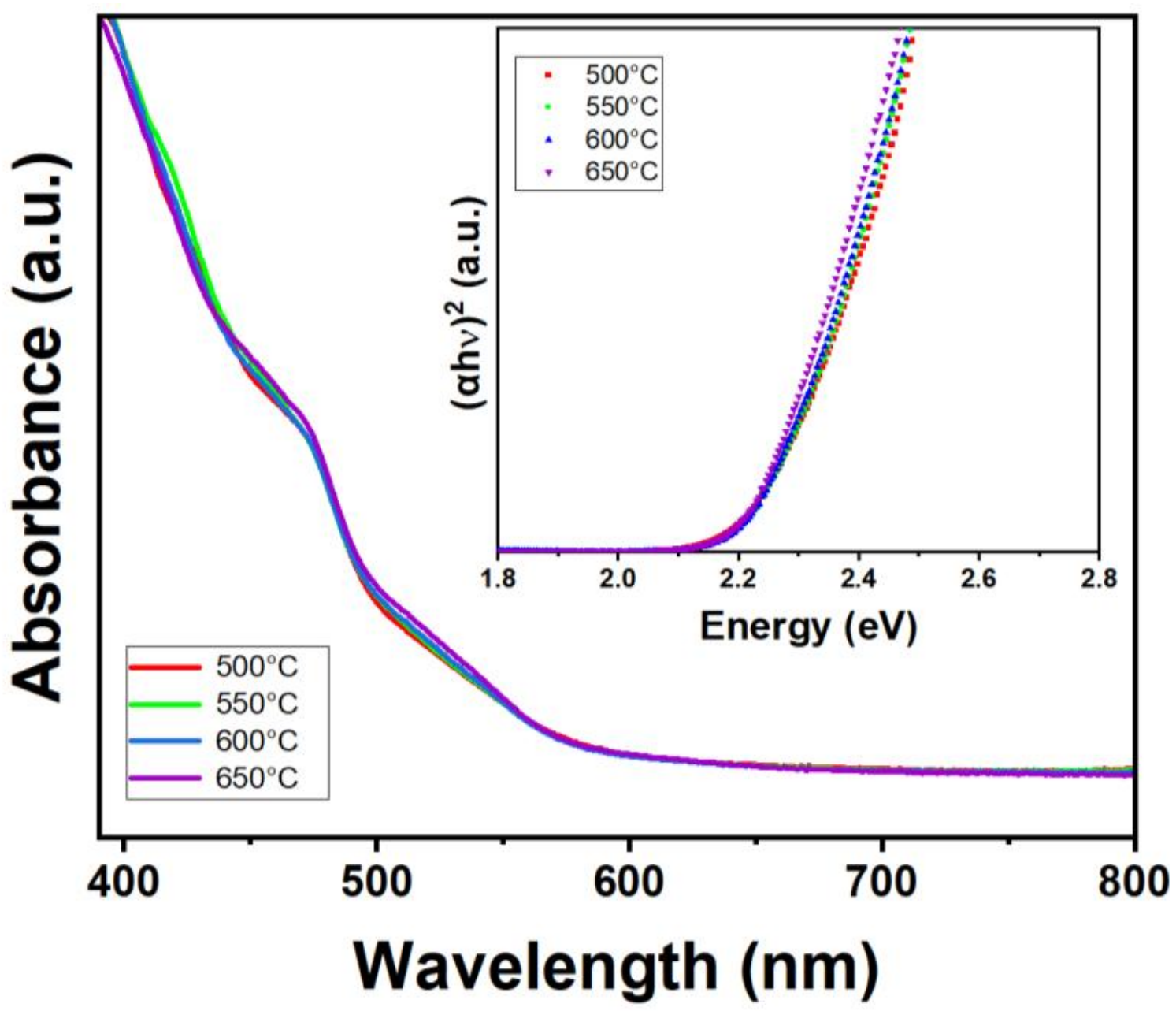

Figure S3. UV–Vis–NIR absorption spectra of air-annealed MFO thin films treated at different annealing temperatures with the corresponding Tauc plots shown in the inset.

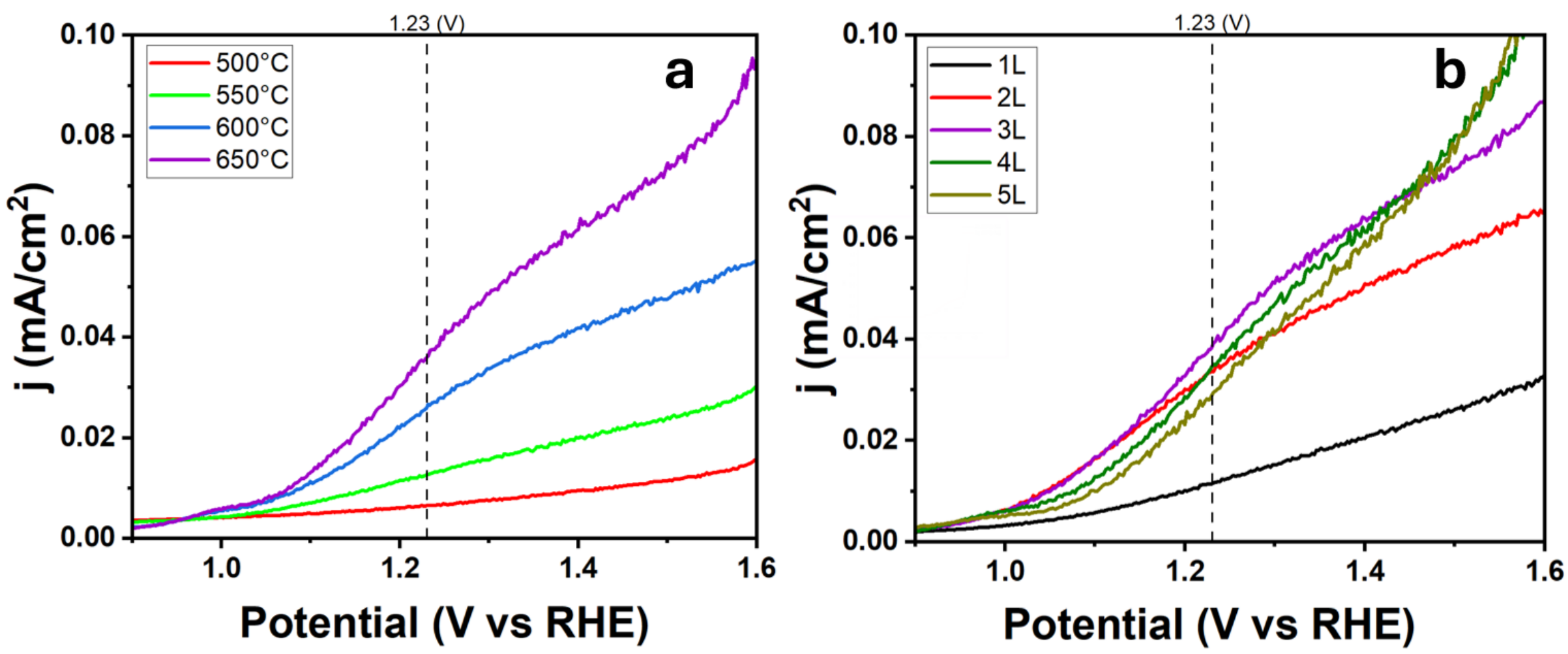

Figure S4. LSV curves for air-annealed MFO thin films measured under simulated sunlight in 1 M KOH: (a) films annealed at different temperatures; (b) films deposited with different numbers of spin-coated layers.

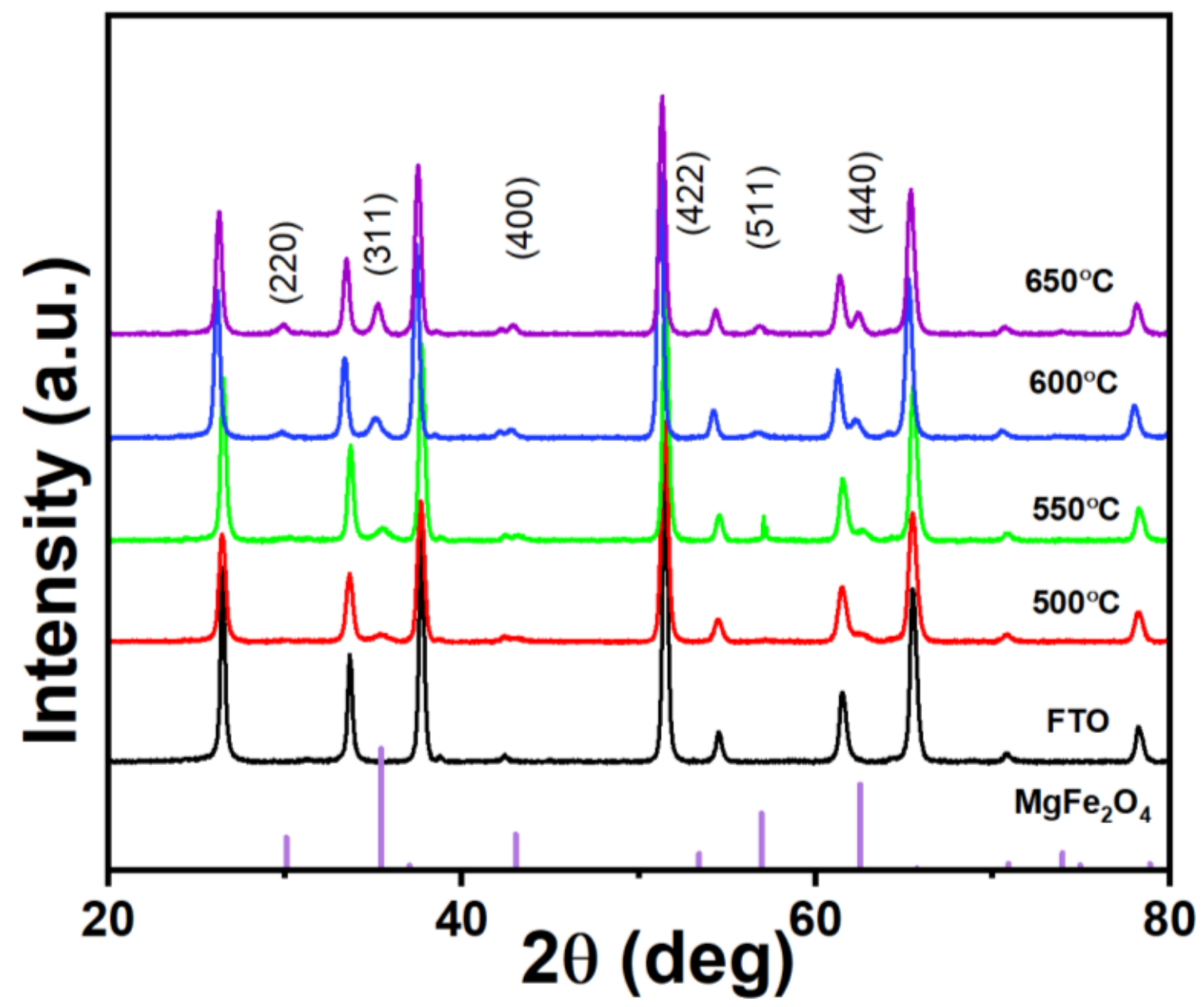

Figure S5. X-ray diffraction (XRD) patterns of MFO thin films annealed in nitrogen at 500, 550, 600, and 650 °C for 2 h (ICDD No. 88-1943).

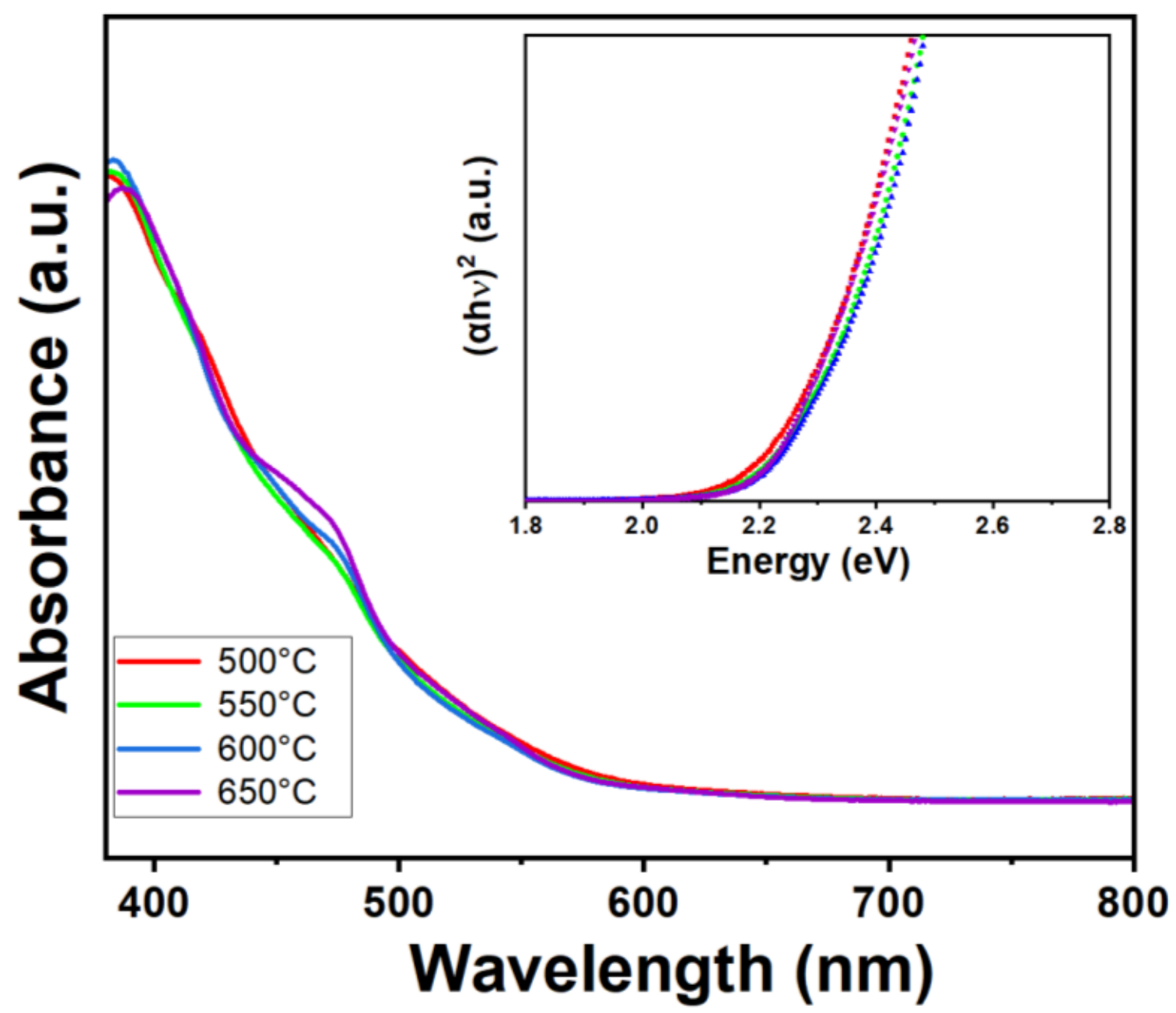

Figure S6. (a) UV–Vis absorption spectra of nitrogen-annealed MFO thin films at different annealing temperatures with the corresponding Tauc plots shown in the inset.

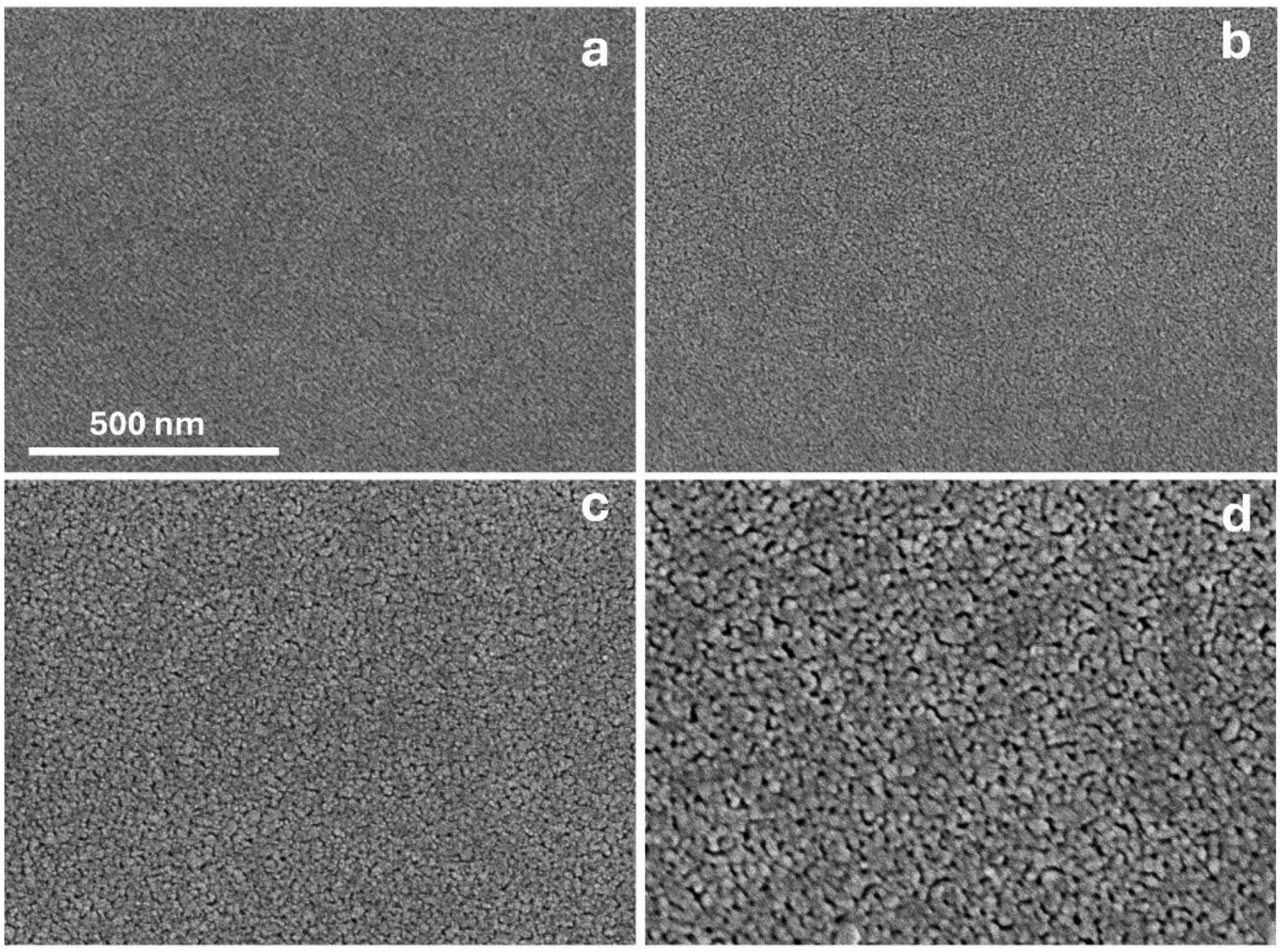

Figure S7. SEM images of nitrogen-annealed MFO thin films at (a) 500, (b) 550, (c) 600, and (d) 650 °C.

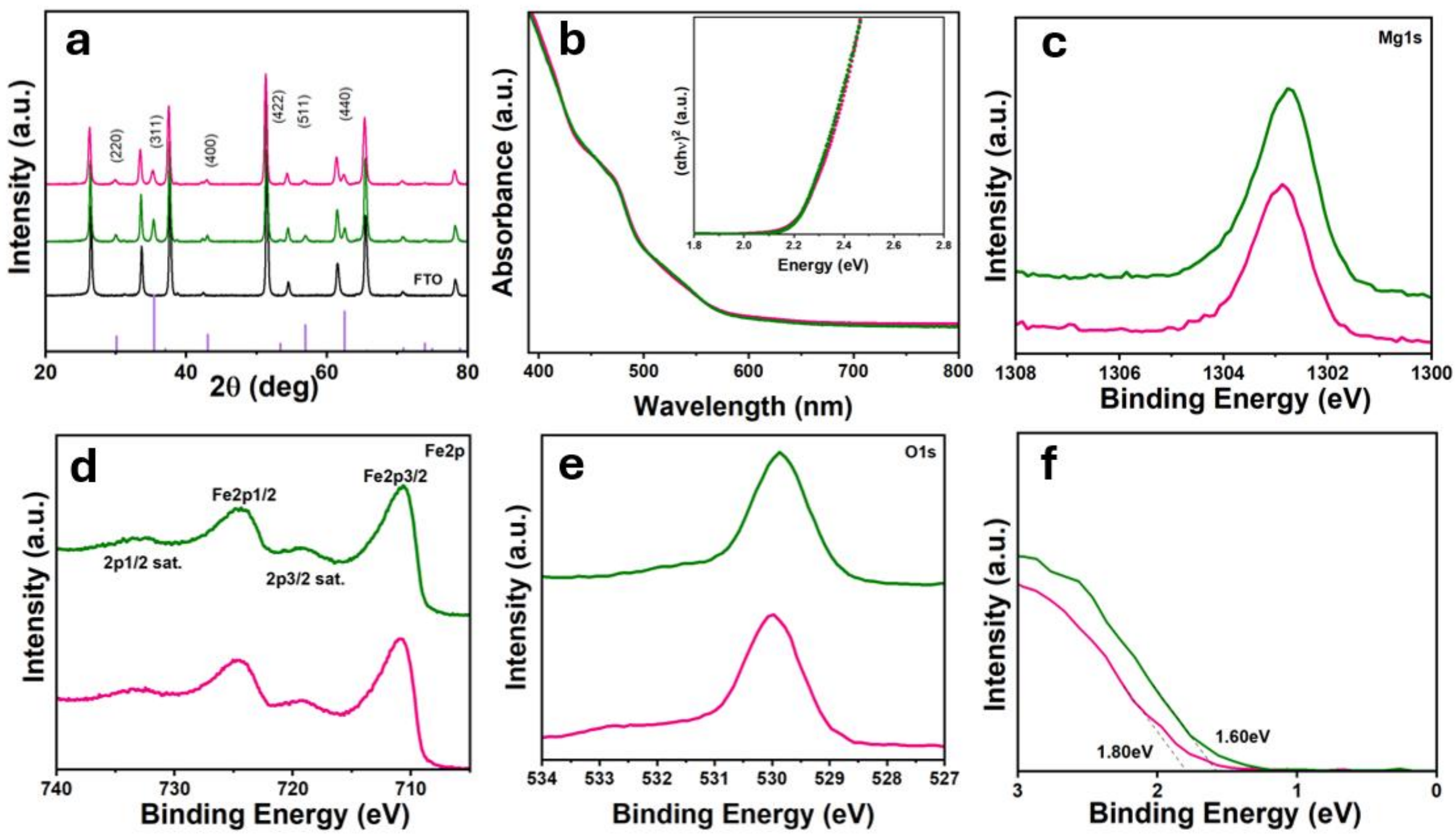

Figure S8. Comparison between air (shown in green) and nitrogen-annealed MFO (shown in pink). a) XRD patterns, (b) UV–Vis absorption spectra (inset: Tauc plots), (c–f) XPS spectra of the Mg 1s, Fe 2p, O 1s and valence regions.

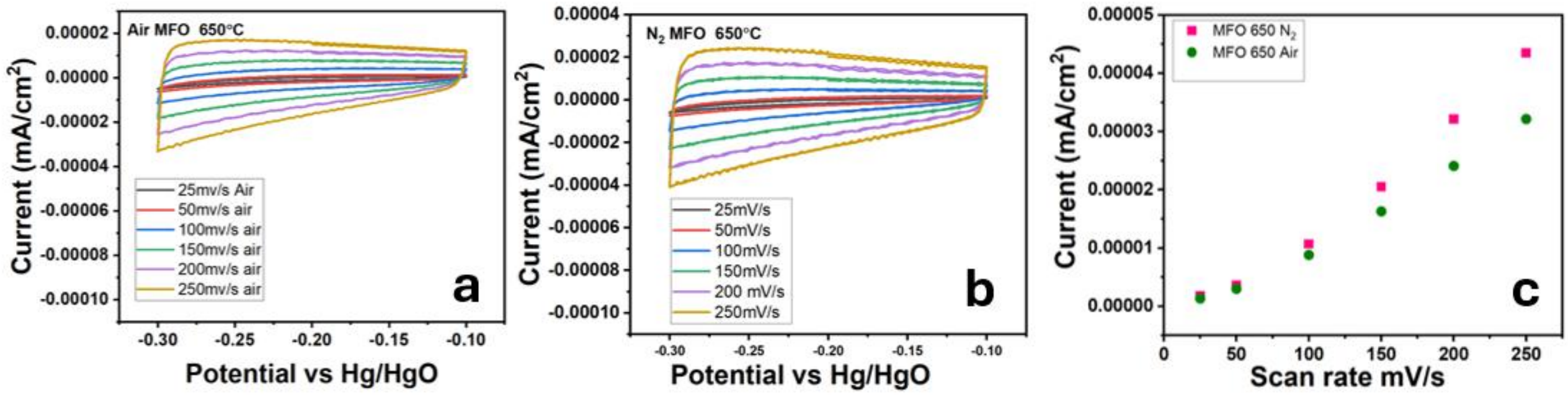

Figure S9. a,b) Cyclic voltammetry (CV) for MFO thin films: (a) air-annealed at 650 °C, (b) nitrogen-annealed at 650 °C. c) Relationship between current and scan rate at -0.20 V vs. Hg/HgO. The slope of these curves is the double layer capacitance, which is directly proportional to the electrochemically active surface area (EASA).

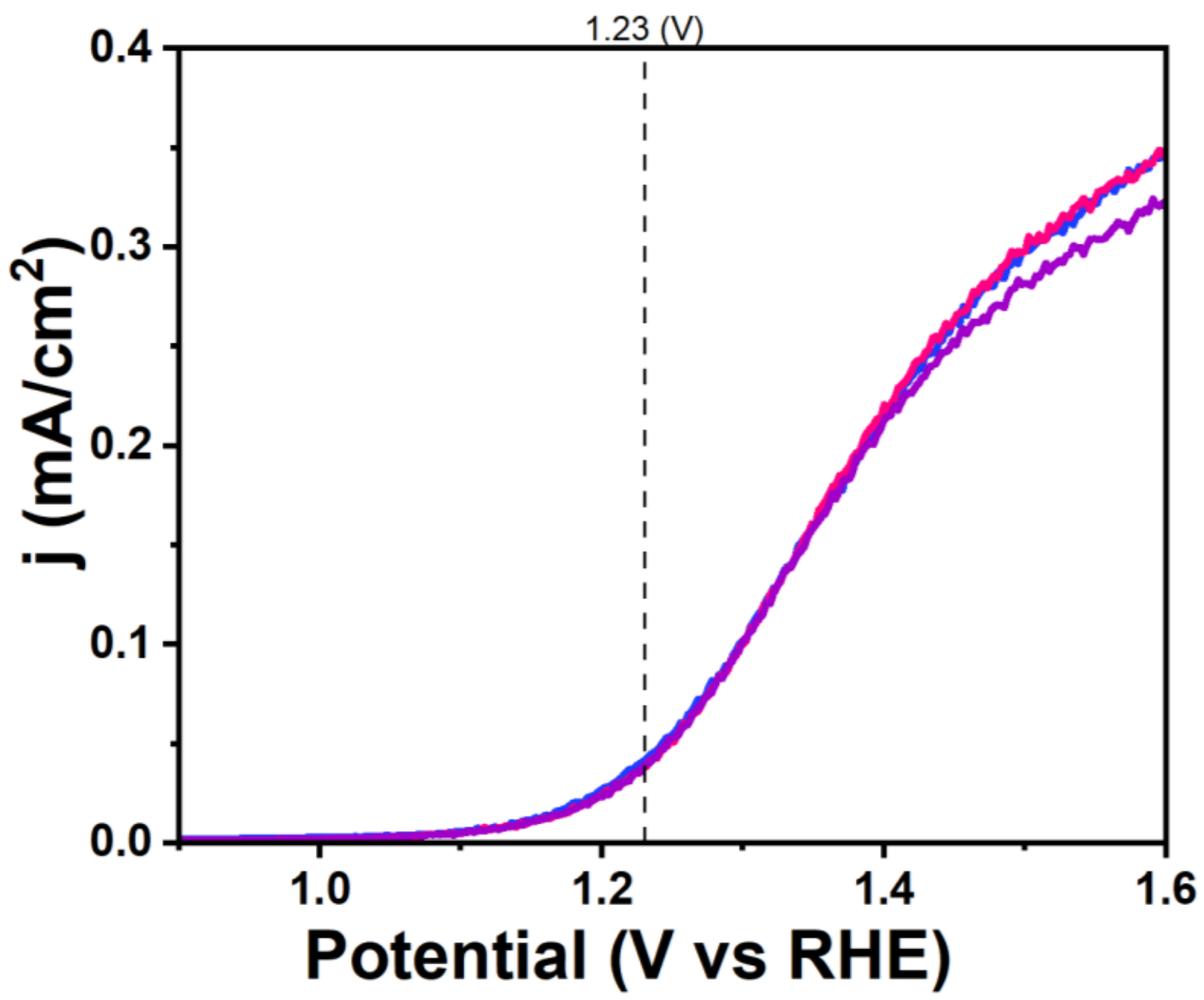

Figure S10. LSV curves for three identical MFO samples (annealed in nitrogen at 650 °C) under simulated sunlight in 1M KOH.

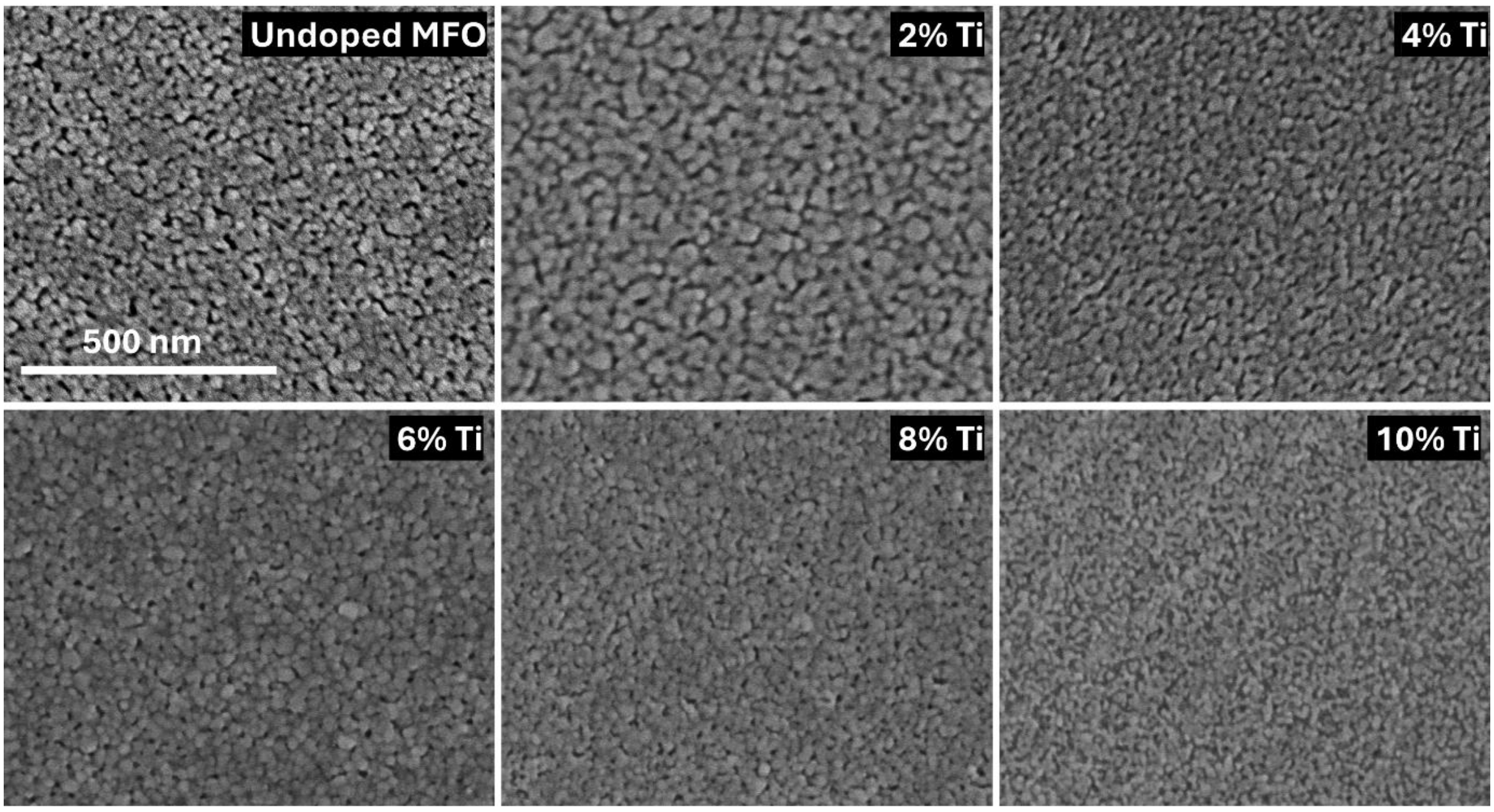

Figure S11. SEM images of undoped and Ti-doped MFO thin films with different Ti concentrations.

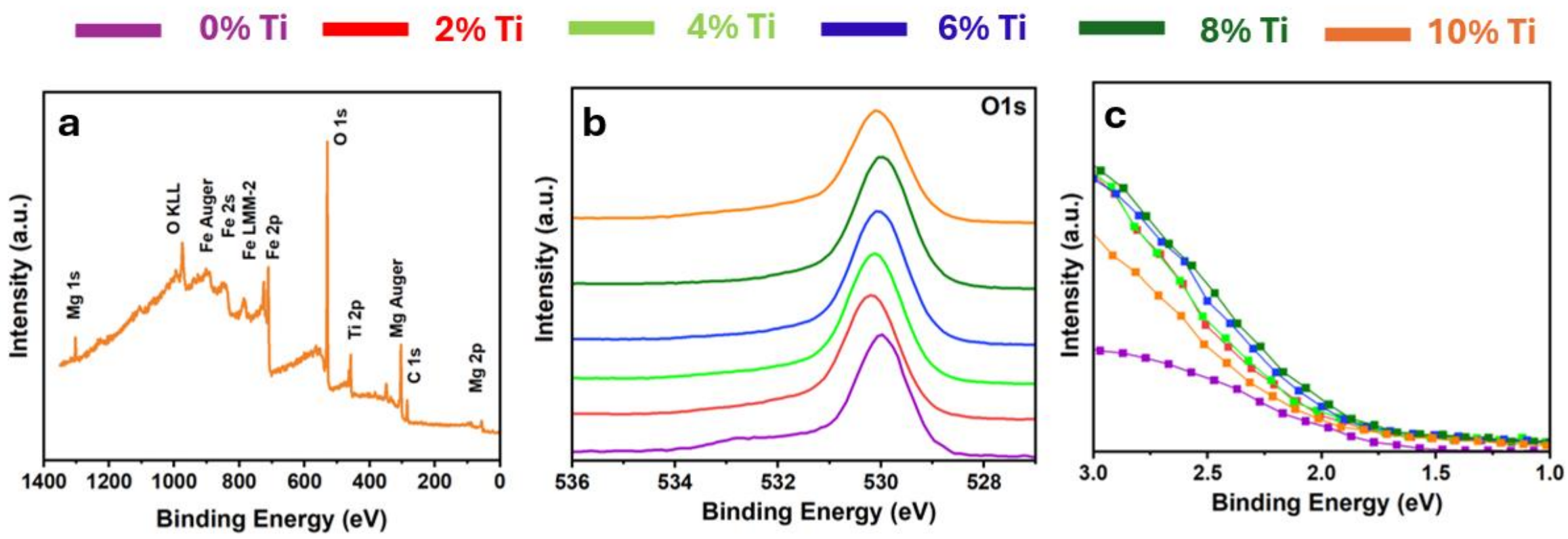

Figure S12. XPS characterization of Ti-doped MFO thin films: (a) survey scan, (b) O 1s region, and (c) valence band region.

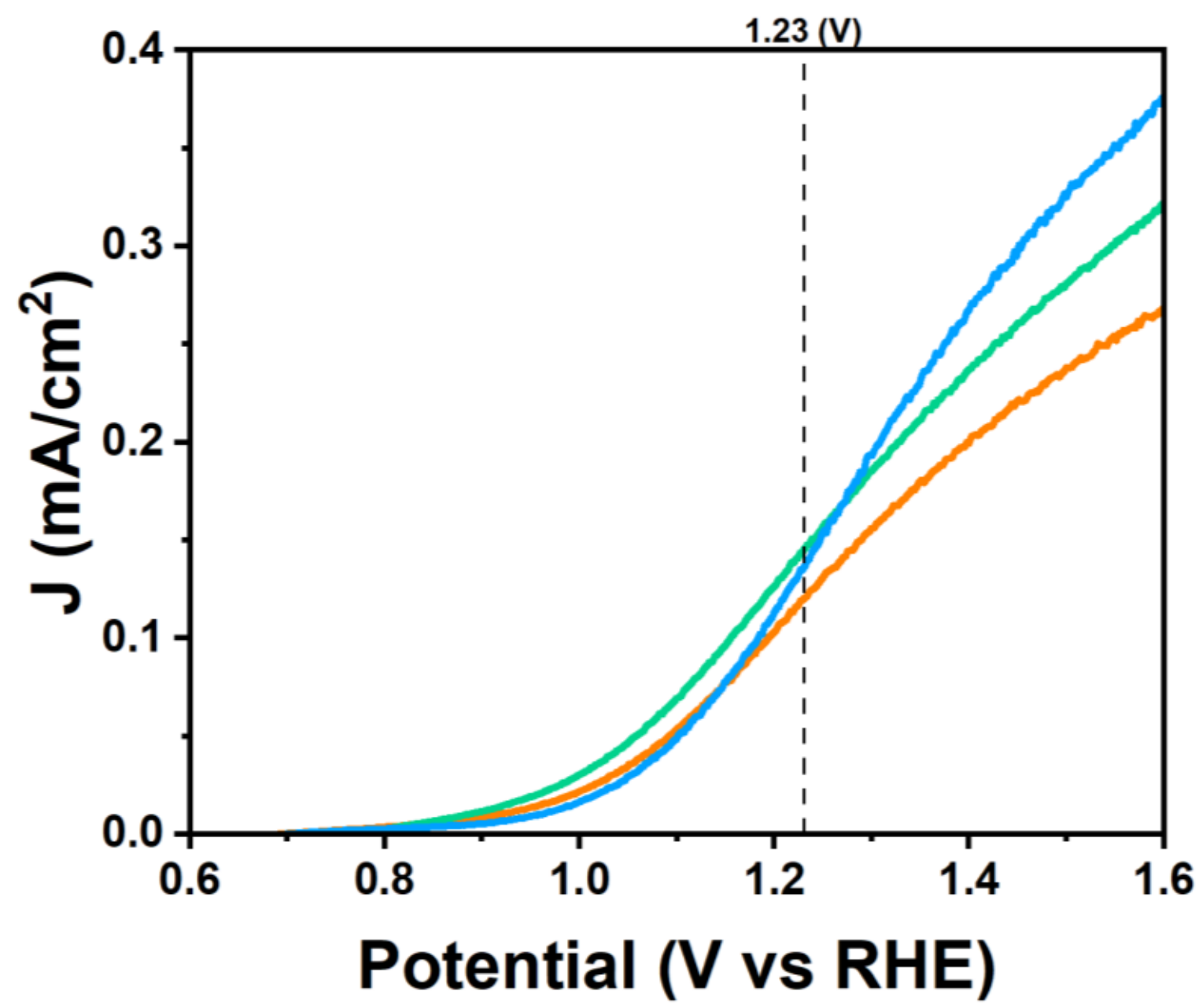

Figure S13. LSV curves for three identical 10 at.% Ti-doped MFO thin films (annealed in nitrogen at 650 °C) under simulated sunlight in 1M KOH.

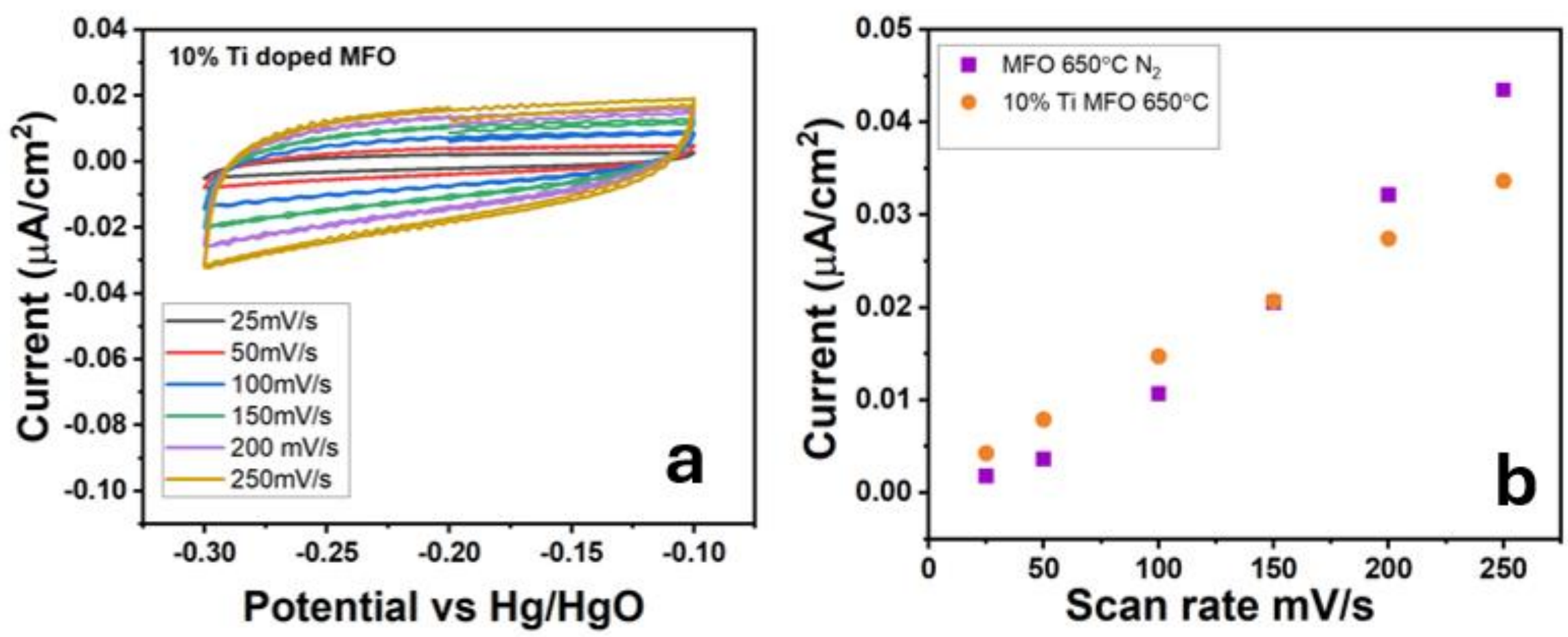

Figure S14. a) CV scan for 10 at.% Ti-doped sample MFO. b) Relationship between current and scan rate at -0.20 V vs. Hg/HgO. The slope of these curves is the double layer capacitance, which is directly proportional to the EASA.

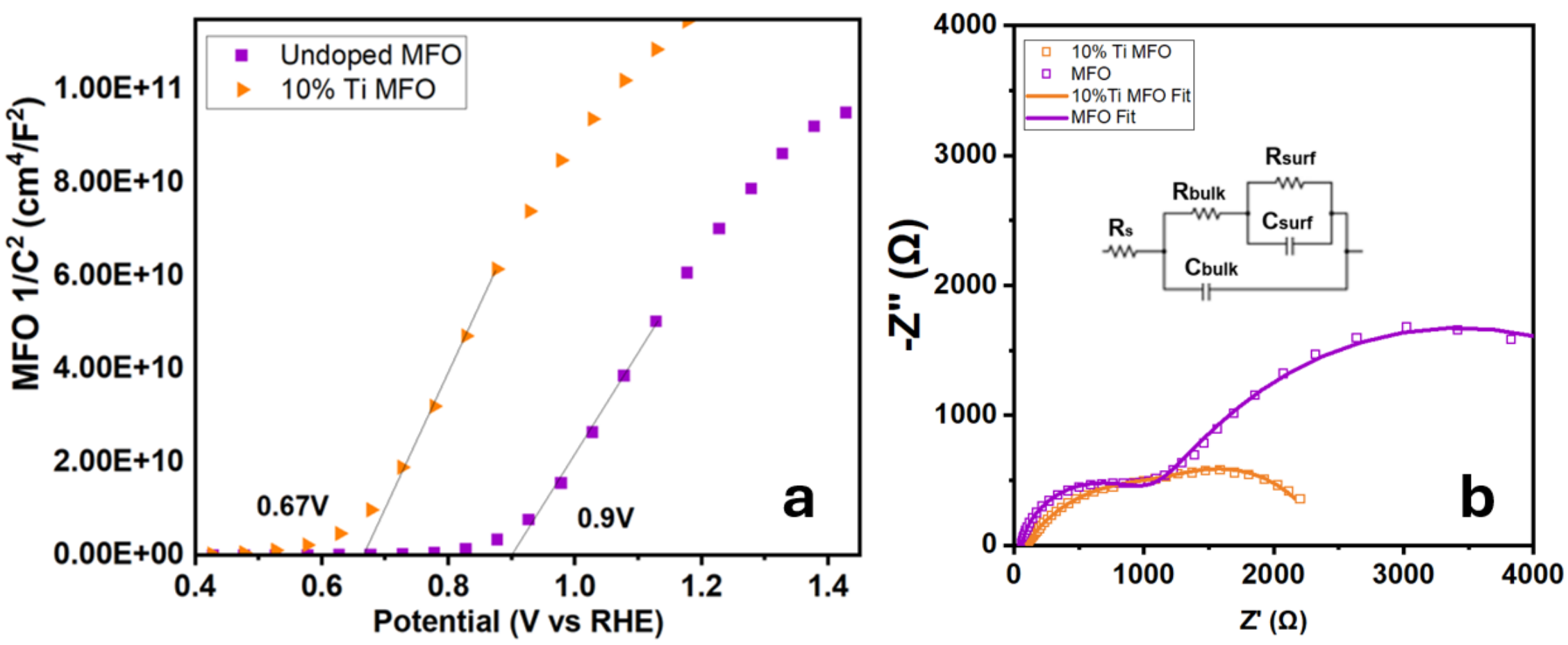

Figure S15. EIS results of undoped and Ti-doped MFO thin films (a) Mott–Schottky plots, (b) Nyquist plots. The inset shows the equivalent circuit used.

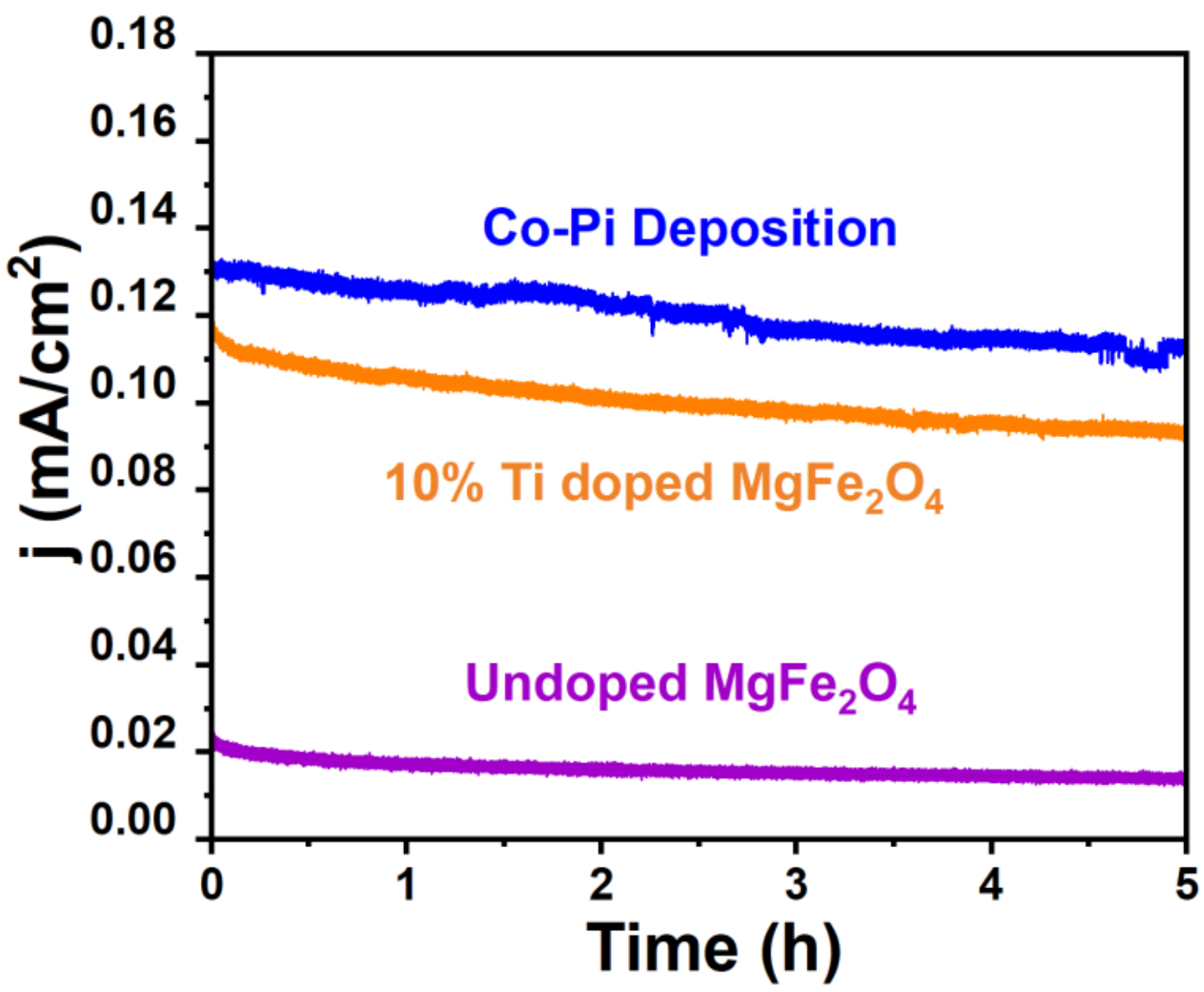

Figure S16. Chronoamperometric stability measurements at 1.23 V vs. RHE for undoped,10% Ti-doped MFO and 10% Ti-doped MFO after Co-Pi deposition for 5 h.

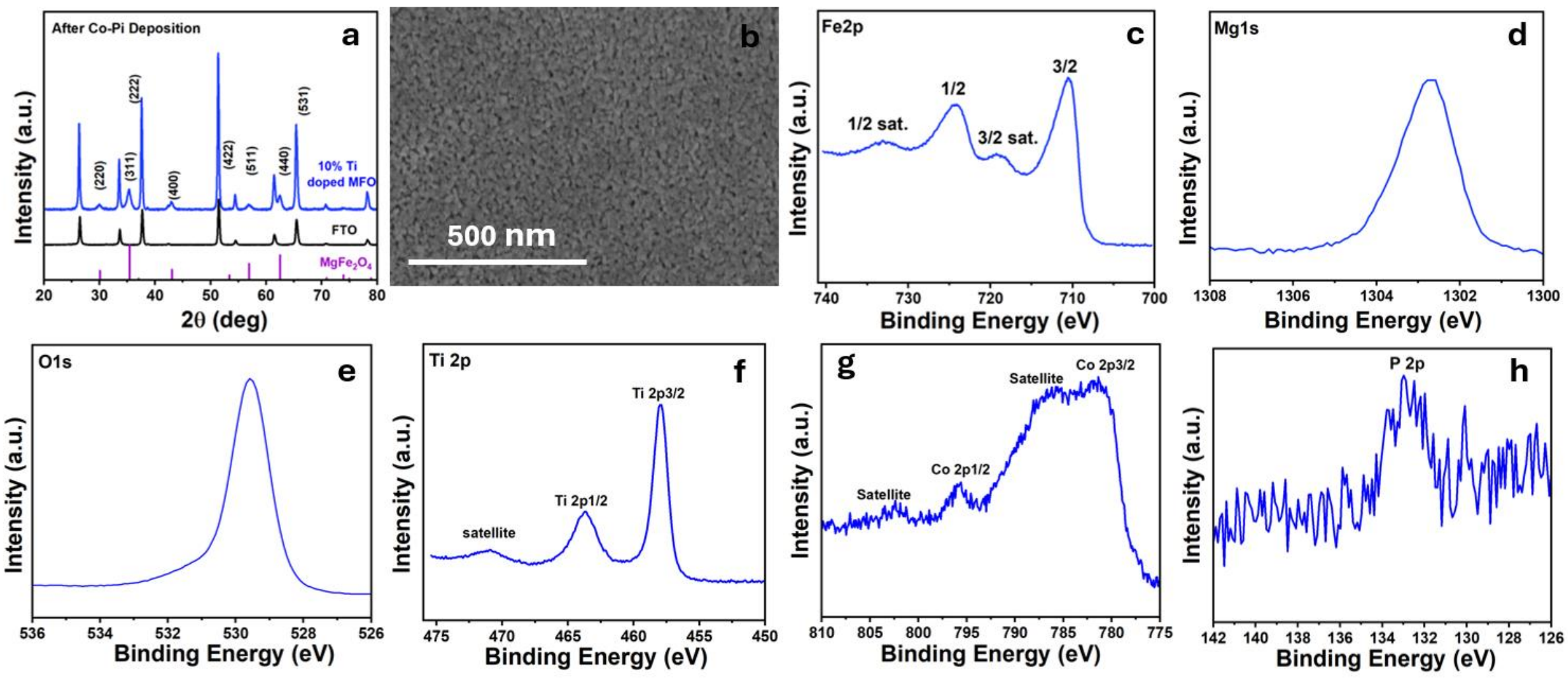

Figure S17. Post-stability characterization of the Co-Pi modified 10 at.% Ti-doped MFO photoanode after 5 h of PEC water oxidation in 1 M KOH (pH 13.7): (a) XRD pattern, (b) SEM image, and high-resolution XPS spectra of (c) Fe 2p, (d) Mg 1s, (e) O 1s, (f) Ti 2p, (g) Co 2p, and (h) P 2p.

Annealing Temperature (°C)	*Crystallite size from the (311) peak (nm)*	*Particle size (nm)*	*Direct band gap (eV)*
500	10.6	15.4 ± 4.7	2.25
550	12.7	13.5 ± 4.1	2.24
600	14.7	22.2 ± 5.2	2.23
650	20.4	23.5 ± 5.0	2.21

Table S1. Crystallite size, particle size and band gap of air-annealed MFO thin films at different annealing temperatures.

Annealing Temperature (°C)	***Crystallite size from the (311) peak (nm)***	***Particle size (nm)***	***Direct band gap (eV)***
500	6.6	/	2.20
550	8.3	/	2.22
600	10.9	/	2.23
650	15.3	13.4 ± 3.4	2.20

Table S2. Crystallite size, particle size and band gap of nitrogen-annealed MFO thin films at different annealing temperatures. Particle size analysis was not possible in some samples due to the inability to distinguish individual particles. Although the crystallite size is slightly larger than the mean SEM particle size for the 650 °C sample, this apparent discrepancy remains within the uncertainty of the SEM measurement and is therefore considered to arise from the experimental challenges in identifying particles boundaries.

Sample	J_{Abs}	$J_{Na_2SO_3}$	J_{KOH}	$\eta_{bulk} = \frac{J_{Na_2SO_3}}{J_{Abs}}$	$\eta_{surf} = \frac{J_{KOH}}{J_{Na_2SO_3}}$
Air annealed 650°C	5.89	0.181	0.0362	3.1 %	19.9 %
N_2 Annealed 650°C	5.92	0.267	0.0451	4.5 %	16.9 %
2% Ti	6.21	0.268	0.0802	4.3 %	29.9 %
4%Ti	6.38	0.285	0.1053	4.5 %	36.9 %
6% Ti	7.32	0.295	0.1090	4.1 %	37.0 %
8% Ti	7.68	0.287	0.1072	3.7 %	37.4 %
10% Ti	7.63	0.338	0.1412	4.4 %	41.4 %

Table S3. charge separation efficiency in the bulk (η_{bulk}) and charge transfer efficiency on the surface (η_{surf}) for selected samples prepared in this study calculated from the photocurrent measured in the presence ($J_{Na_2SO_3}$) and in the absence (J_{KOH}) of the hole scavenger Na_2SO_3, and from the maximum photocurrent density achievable based on the optical absorption of each photoelectrode (J_{Abs}).

Ti doping (at. %)	***Crystallite size from the (311) peak (nm)***	***Particle size (nm)***	***Direct band gap (eV)***	***E_F - E_{VB} (eV)***	***E_{CB} - E_F (eV)***
0	15.3	13.4 ± 3.4	2.22	1.80	0.42
2	15.2	14.2 ± 3.5	2.20	1.81	0.39
4	14.9	15.0 ± 3.6	2.19	1.87	0.32
6	13.6	15.5 ± 2.9	2.12	1.86	0.26
8	11.5	14.5 ± 4.7	2.08	1.82	0.26
10	10.3	14.3 ± 4.5	2.05	1.80	0.25

Table S4. Crystallite size, particle size, band gap and positions of valence band (VB) edge and conduction band (CB) edge relative to the Fermi energy (E_F) for Ti-doped MFO thin films at different Ti concentrations. As per the case in Table S2, the fact that the crystallite size is larger than the SEM particle size in the 0% and 2% Ti samples is due to uncertainties in the identification of particles edges during SEM analyses.
